# Meta-analysis of six dairy cattle breeds reveals biologically relevant candidate genes for mastitis resistance

**DOI:** 10.1186/s12711-024-00920-8

**Published:** 2024-07-15

**Authors:** Zexi Cai, Terhi Iso-Touru, Marie-Pierre Sanchez, Naveen Kadri, Aniek C. Bouwman, Praveen Krishna Chitneedi, Iona M. MacLeod, Christy J. Vander Jagt, Amanda J. Chamberlain, Birgit Gredler-Grandl, Mirjam Spengeler, Mogens Sandø Lund, Didier Boichard, Christa Kühn, Hubert Pausch, Johanna Vilkki, Goutam Sahana

**Affiliations:** 1 Center for Quantitative Genetics and Genomics, Aarhus University, 8000 Aarhus, Denmark; 2 Natural Resources Institute Finland (Luke), 31600 Jokioinen, Finland; 3 Université Paris-Saclay, INRAE, AgroParisTech, GABI, 78350 Jouy-en-Josas, France; 4 Animal Genomics, ETH Zurich, 8092 Zurich, Switzerland; 5 Wageningen University and Research, Animal Breeding and Genomics, P.O. Box 338, 6700 AH Wageningen, The Netherlands; 6 Institute of Genome Biology, Research Institute for Farm Animal Biology (FBN), 18196 Dummerstorf, Germany; 7 Agriculture Victoria, AgriBio, Centre for AgriBiosciences, Bundoora, VIC Australia; 8 School of Applied Systems Biology, La Trobe University, Bundoora, VIC 3083 Australia; 9 Qualitas AG, 6300 Zug, Switzerland; 10 Agricultural and Environmental Faculty, University Rostock, 18059 Rostock, Germany

## Abstract

**Background:**

Mastitis is a disease that incurs significant costs in the dairy industry. A promising approach to mitigate its negative effects is to genetically improve the resistance of dairy cattle to mastitis. A meta-analysis of genome-wide association studies (GWAS) across multiple breeds for clinical mastitis (CM) and its indicator trait, somatic cell score (SCS), is a powerful method to identify functional genetic variants that impact mastitis resistance.

**Results:**

We conducted meta-analyses of eight and fourteen GWAS on CM and SCS, respectively, using 30,689 and 119,438 animals from six dairy cattle breeds. Methods for the meta-analyses were selected to properly account for the multi-breed structure of the GWAS data. Our study revealed 58 lead markers that were associated with mastitis incidence, including 16 loci that did not overlap with previously identified quantitative trait loci (QTL), as curated at the Animal QTLdb. Post-GWAS analysis techniques such as gene-based analysis and genomic feature enrichment analysis enabled prioritization of 31 candidate genes and 14 credible candidate causal variants that affect mastitis.

**Conclusions:**

Our list of candidate genes can help to elucidate the genetic architecture underlying mastitis resistance and provide better tools for the prevention or treatment of mastitis, ultimately contributing to more sustainable animal production.

**Supplementary Information:**

The online version contains supplementary material available at 10.1186/s12711-024-00920-8.

## Background

Mastitis is an inflammation of the mammary gland and udder tissue caused by trauma or infection. As an udder disease, mastitis can affect the production of dairy cows in many ways, including milk yield, milk composition, and milk properties [1–3]. Besides the negative effect on milk yield and quality, mastitis is painful to the animal and often requires antibiotic treatment and, if persistent, may lead to early culling [4]. Meanwhile, both prevention and treatment of mastitis are costly [5]. The outcome of a mastitis infection is influenced by many factors, including the resistance of the host, the pathogen(s) in question, the interaction between pathogen and host, and the environment [6, 7].

Clinical mastitis (CM) incidence and somatic cell count (SCC) are commonly used phenotypes for genetic studies on mastitis. CM is often defined as a binary trait, depending on whether or not a cow shows symptoms of mastitis (e.g., clots, flecks, and change of color and consistency of milk, udder swelling, pain, fever) within a specific lactation interval. SCC is the number of somatic cells, primarily leukocytes, in milk. When infection occurs, leukocyte, and especially neutrophil count in milk strongly increases as an immune response to a mastitis-causing pathogen [8]. Because there is no routine direct measurement of pathogens nor of immune response, SCC is an indirect indicator of mastitis and can be used to detect mastitis before clinical symptoms develop (subclinical). However, SCC also varies depending on many factors other than mastitis, e.g., parity or lactation stage [9]. Somatic cell score (SCS) is the log-transformed SCC [10], which has been commonly used in genetic evaluation as an indicator trait for mastitis [11]. The heritability of CM is low, ranging from 0.02 to 0.12 [6, 7] and its genetic correlation with milk yield is unfavorable, ranging from 0.24 to 0.55 in Nordic dairy cattle [11] and 0.45 in first lactation French Holstein cattle [12]. Heritability of SCS is also low (0.10 to 0.15) [12] but somewhat higher than that of CM, and its genetic correlation with milk yield is less unfavorable than for CM (0.1–0.2) [12–14].

Using single nucleotide polymorphism (SNP) array genotypes, many association studies have been conducted to identify genetic factors affecting SCS, while CM has rarely been considered for such studies. For both traits, a high number of quantitative trait loci (QTL) have been reported in the animal QTL database (AnimalQTLdb, http://www.animalgenome.org/cgi-bin/QTLdb/BT/index, accessed on February 2023) [15]. However, inconsistent findings among studies indicate the complexity of finding causal variants underlying mastitis resistance in dairy cattle.

The AnimalQTLdb [15] contains 1869 and 569 QTL for SCS and CM, respectively [15], with QTL reported on all *Bos taurus* autosomes (BTA) for CM and SCS, except on BTA29 for SCS. Some chromosomes are overrepresented for these traits (BTA6, BTA12, BTA24, BTA25, BTA28), especially for CM [15], however, only a handful of QTL are consistently reported across studies. In a systematic review on 39 selected mastitis QTL studies, Narayana et al. [16] observed little overlap (0.02%) of candidate genes across studies. A QTL for CM and SCS at approximately 88–89 Mb on BTA6 (based on ARS_UCD1.2 assembly [17]) has been reported in many studies, including in Nordic dairy cattle breeds [18–23], in Nordic and Italian Holstein [24], US Holstein [25], German Holstein [26], Italian Valdostana Red Pied [27], and French Holstein and Montbéliarde cows [28]. The best plausible candidate gene for this QTL is the *GC* gene, which encodes Vitamin D-binding protein [19, 22, 23], but earlier results indicate more than one potential causal gene in this QTL region, including the *NPFFR2* gene [22, 23, 28]. In addition, a copy number variant (CNV) in *GC* has been proposed as the potential causal mutation [29]. Information on candidate genes and candidate variants for most identified QTL is still incomplete, as well as knowledge about whether the same QTL segregates across breeds.

Lack of reproducibility of QTL identified for mastitis traits in AnimalQTLdb [15] between different studies and breeds may be due to the complex nature of mastitis resistance (low heritability, effects of environment, variation in the phenotype definition), the segregation of different variants in different breeds, and potential genotype by environment interaction effects. Furthermore, the specific details of separate studies, e.g., different marker densities and variable linkage disequilibrium (LD) structure between breeds, may lead to identification of different QTL intervals, even if the underlying causal variants are the same. Finally, the limited power to identify variants with small effect size may have led to both false negative and false positive reports.

Recently, GWAS/QTL mapping studies have adopted whole genome sequence (WGS) level markers [19, 30], which could facilitate the fine mapping of QTL regions. However, when applied to a limited number of animals and within breeds, these studies have not always yielded great improvement in QTL detection and fine mapping, due to the limited power in the study design or extensive LD in the population. Meta-analyses that combine GWAS summary statistics from multiple studies and breeds could increase both the power and precision to identify genetic variants affecting mastitis-related traits [31]. While GWAS identifies significant genetic markers, it is only the first step in the journey towards a comprehensive understanding of the underlying genetic mechanisms. In contrast to human GWAS, post-GWAS analysis in livestock studies is limited, primarily due to lack of comprehensive annotation information. However, a diverse array of post-GWAS methods and strategies can be employed to connect genomic variants to trait variation in cattle. One such strategy is using phenotype related and tissue specific RNAseq dataset [19, 32] to prioritize candidate genes. Annotation of variants by Variants Effect Predictor (VEP) [33] is another common option used. Gene analysis developed for human studies could also be applied to livestock, as they usually only require a GWAS dataset and gene location information [34]. Recent projects have brought new annotation information of livestock genomes that is useful for post-GWAS analysis, including cattleGTEx [35], FAANG [36], and Bovreg (https://www.bovreg.eu/). However, all these resources and methods need to be fine-tuned together to maximize the chance to pinpoint the causal genes and mutations.

In this study, summary statistics of sequence-based GWAS for CM and SCS were combined with single- and multi-trait meta-analysis methods to identify sequence variants associated with CM, SCS, or both. The total number of animals with phenotypes was 30,689 and 119,438 for CM and SCS, respectively. Various post-GWAS analyses were conducted to prioritize candidate genes and reveal the genetic architecture of mastitis in dairy cattle.

## Methods

### Workflow used in the study

**Fig. 1:**
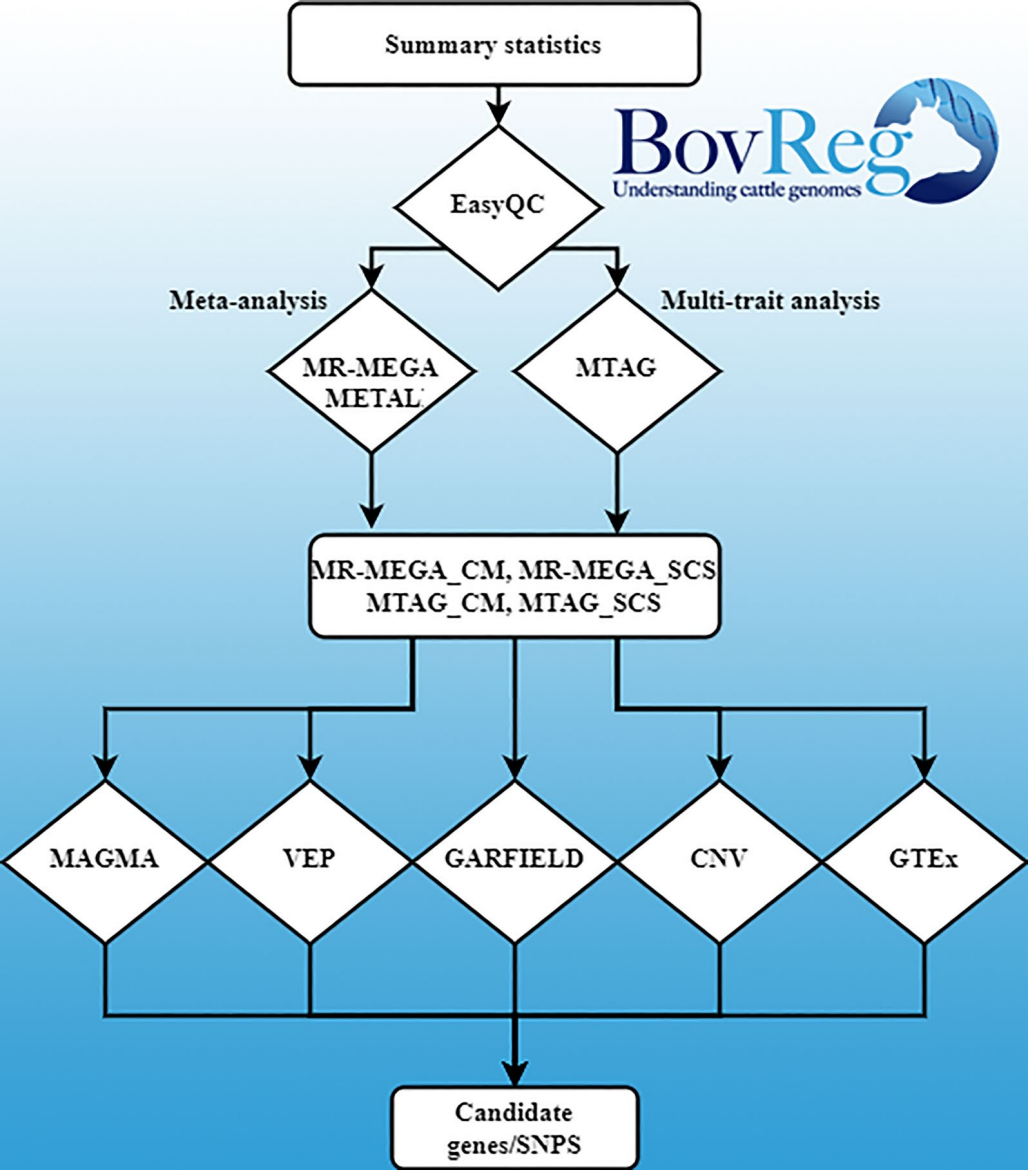
Study workflow. The rectangles show the input data or output result, the diamonds show the analyses performed

We collected GWAS summary statistics from seven partners, including 119,438 records for SCS and 30,689 records for CM. The workflow of the study is illustrated in Fig. 1 and detailed methods are provided below. In summary, we performed quality control at the meta-level across studies using EasyQC [37]. With the clean data, we performed the meta-analysis using a trans-ethnic meta-regression approach implemented in the software MR-MEGA [38], a fixed effect model for meta-analysis using the software METAL [39], and a multi-trait analysis using MTAG [40]. These analyses yielded four outputs, named MR-MEGA_CM for meta-analysis of CM, MR-MEGA_SCS for meta-analysis of SCS, MTAG_CM for multi-trait analysis of CM, and MTAG_SCS for multi-trait analysis of SCS. For post-GWAS, we applied a gene-based analysis using MAGMA [34], annotation of significant variants using Variants Effect Predictor (VEP) [33], genomic feature enrichment using GARFIELD [41], called CNV using an additional dataset, validation with Animal QTLdb and other large-scale GWAS, and checked overlap with the CattleGTEx database [35]. Using all these results, putative causal genes and variants were called. Putative causal genes were called as genes found by nearest gene or gene-analysis that are also supported by gene ontology (GO), Kyoto Encyclopedia of Genes and Genomes (KEGG) pathway, or/and mammalian phenotype database (MPD). Putative causal variants were called as variants located in putative causal genes with support from VEP annotation or/and the key variants from the GARFIELD analysis.

### Imputing sequence variant genotypes

The genotypes from SNP arrays were imputed to WGS level using a two-step imputation approach [42]: first, imputation from a medium density (50 k) SNP array genotypes to the high-density (HD) BovineHD array (approx.777 k) and, second, imputation from the imputed HD genotypes to whole genome sequence variants. All contributors used the same reference genome for imputation, i.e. ARS-UCD1.2 [17] and the WGS reference from the 1000 bull genome project (BGP) Run 7 [43]. The number of animals imputed, size and composition of reference populations, and imputation software used are given (see Additional file 1: Table S1). The map positions of the variants were based on the ARS-UCD1.2-assembly of the *Bos taurus* genome [17].

The dataset included summary statistics from seven partners: Aarhus University (AU), Natural Resources Institute Finland (LUKE), French National Research Institute for Agriculture, Food and the Environment (INRAE), Federal Institute of Technology Zurich (ETH), Research Institute for Farm Animal Biology (FBN), Agriculture Victoria (AgVic), and Wageningen University and Research (WUR).

### Mastitis-related phenotypes

Both traits, SCS and CM, are biologically similar across countries, as shown by the high genetic correlations estimated across countries by Interbull (https://interbull.org/static/mace_evaluations_archive/udder/uappen-014.html). However, the traits are not recorded in the same way in every country and the methods used to define the phenotypes analyzed by GWAS also varied between countries: phenotypes of cows were either mean performances adjusted for environmental effects, i.e., yield deviations (YD), or theoretical phenotypes derived from estimated breeding values and accuracy, i.e., deregressed proofs (DRP); phenotypes of bulls were progeny-based, either DRP or daughter yield deviations (DYD), i.e., mean performance of the progeny adjusted for environmental effects and breeding value of their dam.

In each country, SCC on each test-day was log-transformed to SCS. Final phenotypes were standardized and expressed in genetic standard deviations. The datasets provided by each organization are described in the following.

#### AU/LUKE

Both SCS and CM were analysed in Holstein, Jersey, and Nordic Red breeds. The phenotypes analyzed for association were DRPs of bulls [44]. In Denmark, Sweden and Finland, clinical mastitis was recorded as 0 = no disease or 1 = disease occurred. Records on CM and SCS from 1st to 3rd lactation were used in the genetic evaluation. Separate genetic evaluations were made for Holstein, Red Dairy Cattle (RDC), and Jersey. The model for estimation of breeding values was a multi-trait random regression test-day animal model. See https://www.nordicebv.info for the details.

#### INRAE

Both SCS and CM were analysed in Holstein, Montbéliarde, and Normande breeds. Data originated from the French national database hosted by CTIG at INRAE. Test-day SCS were preadjusted for days in milk and averaged per lactation. Clinical mastitis was defined as 0 (no case) or 1 (at least one case) in the interval between 10 days before and 150 days after calving. For both traits, the genetic evaluation was carried out with an animal repeatability model, considering records from the first three lactations. Bulls DYD were used for association analysis.

#### ETH

DRPs of both SCS and CM were analyzed in Brown Swiss and Original Braunvieh. CM was assessed as a binary trait, i.e., did the cow have at least one mastitis in the interval between 10 days before and 150 days after calving. In addition to the clinical observation, the diagnosis “mastitis” was supported by considering the mean (and standard deviation) SCC until lactation day 150, and the occurrence of a milk sample with > 350,000 somatic cells. Observations from lactations 1 to 5 were considered as repeated measurements. Estimated Breeding Values (EBVs) were estimated using a multi-trait animal model. SCS genetic evaluation was carried out using a test-day animal model. Breeding values were estimated separately for lactations 1, 2, and 3. Lactation-specific traits were weighed equally and aggregated to an average SCS EBV.

#### FBN

SCS from weekly milk samplings of Holstein were corrected for fixed effects using a repeatability model and their means were used as phenotype.

#### AgVic

SCS from Holstein and Jersey phenotypes were DRP derived from a test-day evaluation model. This data was provided from the official DataGene processing of national dairy data in May 2020.

#### WUR

Breeding values for Holstein cows were estimated separately for lactations 1, 2, and 3 or higher using a repeatability animal model on SCS test-day records corrected for fixed effect. Lactation-specific EBV were combined in an SCS index using the following weights: 0.4 EBV_SCS_1st lact_ * 0.3 EBV_SCS_2nd lact_ * 0.3 EBV_SCS_≥3rd lact_.

### Statistical analysis for association between sequence variants and the traits

Genome-wide association analysis was performed within each breed on the imputed whole-genome sequence variants using mixed linear models [31, 45]. Data on cows and bulls were analysed separately and considered as independent information, although they are related. The model included the fixed effect of the variant under examination and a random polygenic effect to account for breed stratification and familial relationships. The genomic relationship matrix (GRM) was constructed using markers from the 50 k chip or imputed HD genotypes [31]. The top principal components (4–5) derived from GRM were included in the model as cofactors in some individual analyses. When de-regressed proofs, estimated breeding values, or DYD were used as phenotypes, weighted analysis based on accuracy was performed. In summary, each partner used GCTA-MLMA [46] to conduct an association study. This involved employing a mixed-model approach $y = 1\mu + bx + g + e$. In this equation, **y** represents the vector of trait values, µ is the population mean, b is the fixed effect of the candidate SNP being tested for association, **x** is the vector of SNP genotype dosages, **g** represents the vector of polygenic effects captured by the GRM with $\textbf {g} \sim N(\textbf {0},{\textbf{G}}{\sigma }_{g}^{2})$, and **e** is the vector of residual errors with $\textbf{e }\sim N(\textbf{0}, {\textbf{I }}{\sigma }_{e}^{2})$, where ${\textbf{G}}$ is the GRM by the default setting of GCTA, ${\textbf{I}}$ is identity matrix, ${\sigma }_{g}^{2}$ is the genetic variance and ${\sigma }_{e}^{2}$ is the error variance. The SNP with the smallest p-value within a peak was assigned as the lead SNP.

### Summary statistics quality control

Before meta-analysis, we performed the following quality control on summary statistics from each partner in order to check inconsistencies, for example, to identify problems with allele frequencies or strand, level of breed stratification (lambda values) etc. First, we removed imputed variants with minor allele frequency (MAF) < 0.5% and imputation accuracy < 0.4, as assessed by the R^2^ given by the different imputation software programs. Then, the variant effect estimates were standardized by dividing them by the genetic standard deviation of the trait, as provided by each partner for each breed and trait. Variants with effect size estimates larger than three times the genetic standard deviation were removed. Lastly, we applied EasyQC on the filtered data following the protocol from EasyQC’s developer [37].

### Meta-analysis

We applied two meta-analysis methods to the clean summary statistics data, a fixed effect model for a meta-analysis using the software METAL [39] and a trans-ethnic meta-regression approach implemented in the software MR-MEGA [38]. For METAL, we followed recommendations from EasyQC’s protocol [37]. Briefly, we ran METAL with the fixed effects method using the STDERR command, which weights effect size estimates using the inverse of the corresponding standard errors and set parameter AVERAGEFREQ to report the average allele frequency and set parameter MINMAXFREQ to report the minimum allele frequency across all files. For MR-MEGA, we used the default setting to run the meta-analysis but used PCs in the linear regression model to estimate the reference allele effect across GWAS. We used 4 PCs for CM and 12 PCs for SCS, based on the MR-MEGA guideline for the number of PCs to fit to equal the number of populations minus 2.

### Multi-trait meta-analysis

We used the MTAG software [40], which can take account of sample overlap, to perform the multi-trait analysis for each breed with summary statistics for both CM and SCS and then combined the output afterwards with MR-MEGA, which can account for heterogeneity in allelic effects. The same significance threshold applied for single trait analysis was also applied for the multi-trait meta-analysis as − log10(p) > 8.5.

### Comparing the QTL

To identify novel QTL, we used two sets of comparison: (1) the lead SNPs of MR-MEGA_CM and MR-MEGA_SCS were directly compared to the lead SNPs from single analysis and if the distance of two lead SNPs was less than 2 Mb, we considered them as overlap QTL, otherwise, we defined them as different QTL; (2) the 1 Mb up and down-stream region of the lead SNPs from MR-MEGA_CM, MR-MEGA_SCS, MTAG_CM, and MTAG_SCS were compared to the QTL intervals of the same trait in Animal QTLdb [15] and if there was not overlap, we consider them as different QTL.

### Gene-based analysis

We ran two types of gene-based analyses: MAGMA [34] and eQTL [35]. To setup the database for MAGMA, we generated the gene location file using gene coordinate information from the bed file downloaded from Ensembl v104 [47]. To run MAGMA, we used --gene-model *multi* = *snp-wise* to test mean SNP associations, test top SNP associations, and to combine these two p-values into an aggregate p-value. We also used *--gene-settings adap-permp* to enable adaptive permutation with a maximum of 1,000,000 permutations and a stopping criterion of 10. In addition to the MAGMA analysis, the significant SNPs from single trait meta-analyses and the multi-trait meta-analysis were queried against the significant SNPs (FDR < 0.05) in a recently published eQTL analysis study (https://cgtex.roslin.ed.ac.uk/) [35]. The SNPs which were significant both in the current study and eQTL dataset were retained as potential regulatory variants with effect on udder health. The significant genes were queried against GO [48], KEGG, and MPD [49].

### Variant annotation

Functional consequences of variants were annotated by VEP with cache files (version 104) for combined annotation of Ensembl and Refseq transcripts. To help interpret coding variants, SIFT scores were predicted. By default, the maximum distance to define an up-stream and downstream variant to a gene is 3 Kb. We extracted the annotation of all significant SNPs within a 1 Mb region from the lead SNPs and looked for variants that were predicted by VEP to change the coding sequence or to be located within the regulatory elements.

### Structure variant calling and estimation of linkage disequilibrium

We called structural variants (SV) on chromosome 6 with WGS data from 567 animals that were generated in a previous study [43], which included 123 Nordic Holstein, 60 Jersey, 175 Nordic Red Dairy cattle, and 209 cattle from various other breeds, with approximately tenfold coverage. The raw reads were subjected to Trimmomatic 0.38 [50] with recommended parameters to remove adapter sequence and to trim low-quality bases. Then, the clean reads were mapped to the cattle reference genome ARS-UCD1.2 [17] using bwa mem [51] with parameter ‘M’ to mark shorter split hits as secondary. BaseRecalibrator was applied to the raw alignment bam files using GATK 3.8 [52]. We applied the smoove (https://github.com/brentp/smoove) pipeline to call SVs using the base recalibrated bam files with default parameters by restricting the location to chromosome 6. Then we extracted the SVs of the 123 Nordic Holstein from the joint calls of various breeds. Meanwhile, we extracted the short variants (SNPs and INDELs) of the same individuals from Run8 of 1000 BGP and combined their genotypes with called SVs. To identify *GC* CNV that were found in a previous study [29], we compared the location and length of the CNV we found with the one from the previous study and identified a similar CNV with 3 bp difference and a length of 12 kb CNV. To infer the copy number of the *GC* CNV, we calculated the fold-change for the CNV by depth relative to other regions on the same chromosome using duphold (https://github.com/brentp/duphold), with the resulting value doubled and rounded to obtain the copy number. We assigned the genotype to CNV following [29] and treated 3 copies as 2 copies (wild type) as no cases with 3 copies were reported in [29] and it could be an estimation error due to lower coverage in our dataset compared to the previous study [29]. The previous study [29] showed incorrect calling of flanking genotypes due to the imbalance of the reference and alternative alleles due to the higher copy number of one of the alleles. Thus, we also edited the genotype to change the homozygous alternative allele genotype to a heterozygous genotype for a SNP when there was at least one read support for the reference allele. Finally, we estimated the LD for 72 common Nordic Holstein cattle for both the *GC* CNV and SNP genotypes within a window of 1000 Kb around the *GC* CNV.

### Genomic feature enrichment analysis

We applied a genomic feature enrichment analysis for CM and SCS using GARFIELD [41]. Data were generated in three steps for input into GARFIELD. (1) LD files: low R^2^ (≥ 0.1) to ensure independence of the variants to be tested, and high R^2^ (≥ 0.8) for further annotation of variants. These two files were obtained by Plink using Holstein animals from Run8 of the 1000 BGP animals (closely related individuals were removed) [31]. (2) MAF_TSS distance file: the minor allele frequency (MAF) of Holstein animals from Run8 of 1000 BGP was extracted; the transcription start site (TSS) distance was calculated by bedtools by finding the closest distance between the SNP location and the TSS, as annotated in Ensembl v104 [47]. (3) Annotation file: the regulator elements and CpG islands annotation file were reformatted for GARFIELD. The predicted regulatory elements from ATAC-seq, H3K4me3, H3K27me3, H3K27ac, H3K4me1, and CTCF were retrieved from a recently published data set [53]. We also included CpG islands from the UCSC genome browser. Then, we defined all annotation as genomic features to perform the enrichment analysis and prioritize the possible candidate variants by estimation of the key variants that drive the enrichment features.

## Results

### General information about studies involved

The GWAS summary statistics in this study comprised CM and SCS association tests with imputed whole genome sequence variants following harmonized analysis procedures (see Additional file 1: Table S1) from seven partners. These association studies were conducted in bulls (female progeny test data) and cows from six dairy breeds. The number of bulls and cows submitted by each study participant ranged from 134 to 55,547 individuals by trait and breed group (see Additional file 1: Table S2). Overall, within-breed GWAS were conducted with 119,438 records for SCS and 30,689 records for CM from 68,441 progeny tested bulls and 81,686 cows. Phenotypes were obtained using different approaches (see methods). For cows, the phenotype reflected repeated own performances adjusted for environmental effects and averaged within and across parity. For bulls, phenotypes reflected the average performances of their daughters and were therefore much more accurate than phenotypes for cows, especially for CM, which has very low heritability. The number of genome-wide significant SNPs (− log10(p) > 8.5) discovered in the separate participant studies prior to the meta-analysis ranged from 0 to 2268 (see Additional file 1: Table S3). During quality control of summary statistics, we observed consistency in the direction of allele effect estimates in each breed for most of the significant SNPs (see Additional file 2: Figure S1). The filtered data showed no genomic inflation in any of the studies (see Additional file 1: Table S4 and Additional file 2: Figure S2). For each single-breed GWAS, we checked the concordance of the reported GWAS p-value with the p-value calculated from z-score (see Additional file 2: Figure S3). After quality control, the number of SNPs for meta-analysis ranged from 11,132,286 to 17,134,891 between the studies (see Additional file 1: Table S5). Results from multi-breed meta-analysis and multi-trait analysis were used to pinpoint candidate genes. We collected datasets from publicly available annotations for protein coding sequence and regulatory elements (Fig. 1) to postulate biological connections of association results from meta-analyses with CM and SCS.

Meta-analyses were performed using three approaches. The first two approaches were applied for each trait, while the third approach was a multi-trait analysis: (1) the fixed effects method from the METAL software package [39], (2) the method for multi-ethnic meta-analysis implemented in MR-MEGA [38], and (3) we first applied multi-trait analysis in each breed with summary statistics for both CM and SCS using MTAG [40] and then combined the results from all breeds using MR-MEGA to yield the multi-trait meta-analysis. We chose the MR-MEGA approach to combine multi-trait results because this method showed higher power to detect association signals compared to METAL (results shown below). Henceforth, we named the result of meta-analysis by combining the software and the trait, for example, analysis with MR-MEGA was named MR-MEGA_CM for CM and MR-MEGA_SCS for SCS. The result of the multi-trait meta-analyses were named MTAG_CM for CM and MTAG_SCS for SCS, since MTAG estimated the multi-trait effect and p-value for both traits.

### The meta-analysis for clinical mastitis

**Fig. 2:**
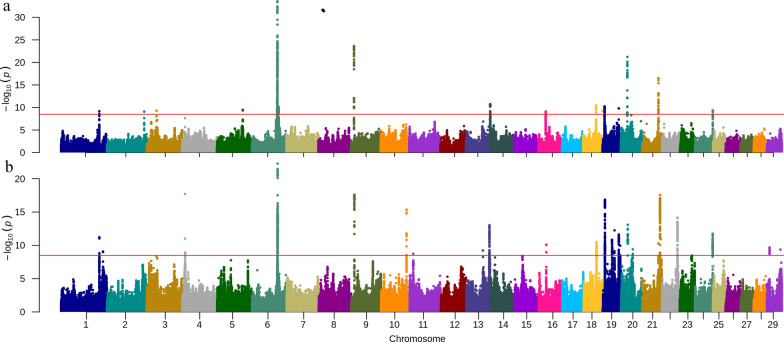
Results for meta-analyses using MR-MEGA approach [38]. **a** Manhattan plot for clinical mastitis . **b** Manhattan plot for somatic cell count . The red horizontal line indicates the genome-wide significance level [− log10(p) = 8.5]

The METAL_CM analysis only detected the well-known locus for CM on BTA6 (see Additional file 2: Figure S4) but breed-specific significant loci from the single breed GWAS were lost (data not shown). Instead, the result from MR-MEGA_CM, which accounts for heterogeneity in allelic effects correlated with ancestry, showed persistence of more within-breed signals and stronger association signals (Fig. 2a). The MR-MEGA_CM analysis detected 15 association signals on 13 autosomes (Table 1 and Additional file 1: Table S6). Consistent with previous studies [18–20, 22], the most significant signal was located on BTA6 at 86,940,863 bp (rs210373936) and is an intergenic SNP close to the *GC* gene. The second strongest association signal was found on BTA9 at 10,457,304 bp (rs133164262), with an intergenic variant near bta-mir-30f as the lead SNP is. The third strongest association signal was located on BTA20, with BTA20: 22,386,425 (rs380944374) as the lead SNP, which is an intron variant of *MAP3K1*. At most one QTL was identified on a chromosome, except for BTA18 and BTA19, which had two identified QTL.

**Table 1:** Lead SNPs for QTL for clinical mastitis (CM) and somatic cell score (SCS), along with the nearest gene and the functional annotation of the SNPs

BTA	Base pair	QTL	rsid	− log10(p)	Analysis method	Nearest gene	Annotation
1	131,393,761	131,144,131–131,643,847	NA	11.23	MR-MEGA_SCS	*SOX14*	Intergenic variant
1	131,399,037	131,149,079–131,649,130	rs209262096	9.15	MR-MEGA_CM	*SOX14*	Intergenic variant
1	131,508,931	131,259,179–131,758,987	rs133645774	19.16	MTAG_SCS	*SOX14*	Intergenic variant
1	143,908,907	143,660,512–144,160,344	rs43282279	9.08	MR-MEGA_SCS	*AGPAT3*	Intergenic variant
1	145,932,212	145,682,433–146,182,333	rs469947398	9.05	MTAG_SCS	*MCM3AP*	Intron variant
2	127,010,295	126,760,970–127,260,998	rs109861338	9.11	MR-MEGA_CM	*TRIM63*	Intergenic variant
2	127,199,316	126,949,469–127,449,526	rs133914262	8.92	MTAG_CM	*PAQR7*	Intergenic variant
3	24,114,904	23,865,017–24,365,480	rs385025933	8.87	MTAG_CM	*TBX15*	Intergenic variant
3	32,937,624	32,688,826–33,188,877	rs43336190	9.29	MR-MEGA_CM	*CYM*	Intergenic variant
3	32,991,793	32,741,957–33,241,801	rs43336135	8.97	MTAG_CM	*PROK1*	Intron variant
3	33,085,262	32,835,778–33,356,862	rs43335461	11.06	MTAG_SCS	*RBM15*	Downstream variant
4	10,207,091	9,958,314–10,457,519	rs211317759	17.71	MR-MEGA_SCS	*ENSBTAG00000051416*	Downstream variant
5	56,290,204	56,040,322–56,542,938	rs109848760	12.68	MTAG_CM	*LRP1*	Intron variant
5	56,332,715	56,082,869–56,583,125	rs208358909	19.19	MTAG_SCS	*STAT6*	Synonymous variant
5	88,406,861	87,673,289–88,657,962	rs209893772	9.50	MR-MEGA_CM	*ABCC9*	Intron variant
6	86,940,863	86,690,963–87,191,548	rs210373936	33.58	MR-MEGA_CM	*GC*	Intergenic variant
6	86,991,630	86,741,866–87,268,801	rs436532576	22.14/19.43	MTAG_CM, MTAG_SCS	*GC*	Intron variant
6	87,000,654	86,752,563–87,280,573	rs108952128	22.27	MR-MEGA_SCS	*GC*	Intron variant
9	10,451,705	10,202,576–10,702,351	rs210770707	17.59	MR-MEGA_SCS	*OGFRL1*	Intergenic variant
9	10,457,304	10,207,394–10,707,412	rs133164262	23.63	MR-MEGA_CM	*bta-mir-30f*	Intergenic variant
9	10,510,020	10,260,034–10,760,190	rs109335443	16.73	MTAG_CM	*OGFRL1*	Intergenic variant
9	10,672,833	10,423,069–10,923,737	rs109718038	25.51	MTAG_SCS	*ENSBTAG00000048046*	Intergenic variant
10	87,184,308	86,811,104–87,436,082	rs42248532	15.37	MR-MEGA_SCS	*TTLL5*	Intron variant
11	12,078,911	11,079,809–12,329,170	NA	8.73	MR-MEGA_SCS	*EXOC6B*	Intron variant
11	85,622,206	85,372,220–85,872,367	rs382615161	10.51	MTAG_SCS	*TRIB2*	Intergenic variant
13	56,890,472	56,012,401–57,140,946	rs41696436	9.22	MR-MEGA_SCS	*PHACTR3*	Intron variant
13	79,482,645	78,816,883–79,738,951	NA	13.03	MR-MEGA_SCS	*ATP9A*	Intron variant
14	528,726	283,062–778,739	rs110418960	9.70	MTAG_CM	*ENSBTAG00000053637*	Intron variant
14	559,962	311,166–885,752	rs210230767	10.75	MR-MEGA_CM	*ADCK5*	Intron variant
16	25,160,034	24,910,289–25,411,234	rs379742673	11.42	MTAG_CM	*DUSP10*	Intergenic variant
16	25,189,022	24,941,012–25,440,400	rs110061124	13.03	MTAG_SCS	*DUSP10*	Intron variant
16	25,190,389	24,953,782–25,460,899	rs109274215	9.12/	MR-MEGA_CM, MR-MEGA_SCS	*DUSP10*	Intron variant
18	43,707,610	43,463,418–43,957,639	rs379699077	10.52	MR-MEGA_CM	*ENSBTAG00000049393*	Intergenic variant
18	44,354,883	43,674,933–44,605,086	NA	10.49	MR-MEGA_SCS	*ENSBTAG00000050669*	Intron variant
18	65,188,613	64,939,546–65,442,597	rs41901751	17.65	MTAG_SCS	*LOC100124497*	Intron variant
18	65,288,692	65,038,910–65,539,395	NA	9.24	MR-MEGA_CM	*ENSBTAG00000038715*	Intergenic variant
19	7,358,480	7,109,618–7,610,867	NA	16.86	MR-MEGA_SCS	*NOG*	Intergenic variant
19	7,371,211	7,122,553–7,622,923	rs109184245	10.25	MR-MEGA_CM	*NOG*	Intergenic variant
19	31,532,436	31,282,440–31,782,555	rs443527170	10.84	MR-MEGA_SCS	*ENSBTAG00000052541*	Intergenic variant
19	40,053,288	39,502,431–40,303,655	rs134402075	12.25	MR-MEGA_SCS	*PGAP3*	Intron variant
19	54,626,232	54,376,382–54,900,608	rs134693405	11.63	MR-MEGA_SCS	*SEPTIN9*	Intron variant
19	56,012,401	55,763,067–56,483,191	rs135790765	9.81	MR-MEGA_CM	*LLGL2*	Intron variant
20	22,385,791	21,933,562–22,635,895	rs110323061	13.09	MR-MEGA_SCS	*MAP3K1*	Intron variant
20	22,386,425	22,141,590–22,636,468	rs380944374	21.22	MR-MEGA_CM	*MAP3K1*	Intron variant
20	22,422,299	22,172,873–22,673,171	rs209103569	19.42	MTAG_CM	*MAP3K1*	Upstream variant
20	22,428,455	22,180,220	rs208280837	15.14	MTAG_SCS	*MAP3K1*	Intergenic variant
20	39,502,431	39,252,645–39,766,694	rs42663967	9.40	MR-MEGA_SCS	*RAI14*	Intron variant
21	57,144,704	56,895,032–57,394,726	rs378255940	16.51	MR-MEGA_CM	*SLC24A4*	Intron variant
21	62,941,833	62,691,932–63,192,091	rs136844062	17.55	MR-MEGA_SCS	*5S_rRNA*	Intergenic variant
21	63,051,720	62,801,824–63,302,337	rs133524129	12.53	MTAG_SCS	*BCL11B*	Intergenic variant
22	52,947,790	52,698,116–53,199,688	rs135845151	14.17	MR-MEGA_SCS	*LTF*	Upstream variant
22	53,007,168	52,757,454–53,257,174	rs385393172	8.58	MTAG_SCS	*CCRL2*	Intron variant
23	39,530,196	39,295,040–39,780,965	rs136857507	13.02	MTAG_SCS	*KIF13A*	Intron variant
24	60,882,420	60,633,040–61,132,920	rs41571207	11.74	MR-MEGA_SCS	*ZCCHC2*	Intergenic variant
24	60,883,696	60,633,830–61,133,857	NA	9.37	MR-MEGA_CM	*ZCCHC2*	Intergenic variant
25	38,531,214	37,561,390–38,781,883	rs383719916	8.54	MTAG_SCS	*LOC618542*	Intron variant
29	9,486,040	9,236,161–9,736,089	NA	9.68	MR-MEGA_SCS	*PICALM*	Intergenic variant
29	46,577,859	46,074,605–46,834,864	rs378268227	9.36	MR-MEGA_SCS	*ENSBTAG00000050252*	Intergenic variant

The list of lead SNPs, their annotation and nearest genes for all the meta-analyses used and implemented in MR-MEGA [38] and MTAG [40]. Suffix “_CM” and “_SCS” are used for CM and SCS respectively

Compared to single breed GWAS, the MR-MEGA_CM analysis helped to identify six new QTL on BTA5, BTA14, BTA18, BTA19 (two on BTA19), and BTA24 (at position 60,883,696). We identified nine novel QTL that were not previously reported in the Animal QTLdb (release 49) [15], located on BTA1, BTA2, BTA3, BTA5, BTA9, BTA16, BTA18: 25 Mb, BTA19: 7 Mb, and BTA21.

### The meta-analysis for somatic cell score

Similar to the meta-analysis for CM, we observed an increase in the number detected QTL by using MR-MEGA (Fig. 2b) compared to METAL (see Additional file 2: Figure S5) for SCS. The MR-MEGA_SCS analysis detected 22 QTL on 15 autosomes (Table 1). Among these, chromosomes BTA1, BTA13, BTA20, and BTA29 each had two QTL and BTA19 had four QTL. Like MR-MEGA_CM, the strongest signal for SCS was located on BTA6 but with a different lead SNP, BTA6: 87,000,654 (rs108952128), which is an intron variant of the gene *GC*. The second strongest signal was located at BTA4: 10,207,091 (rs211317759), which was a downstream variant of ENSBTAG00000051416. The third strongest association was located on BTA9: 10,451,705 (rs210770707), with an intergenic variant near *OGFRL1* as the lead SNP. The lead SNP for the fourth strongest association signal was BTA21: 62,941,833 (rs136844062) and was an intergenic variant near *5S_rRNA*.

The QTL identified around BTA1:131.4 Mb, BTA6:87.0 Mb, BTA9:10.5 Mb, BTA16:25.2 Mb, and BTA20:22.4 Mb were consistent between the meta-analysis and the single breed analyses. The other QTL were novel loci identified by MR-MEGA_SCS compared to single breed analyses. However, at the same time, 11 QTL that were detected in the single breed analyses were not significant in the meta-analysis. By comparing it to the AnimalQTLdb [15], we identify four novel QTL, which are located on BTA1:143 Mb, BTA9, BTA10, and BTA22.

### Multi-trait meta-analysis

**Fig. 3:**
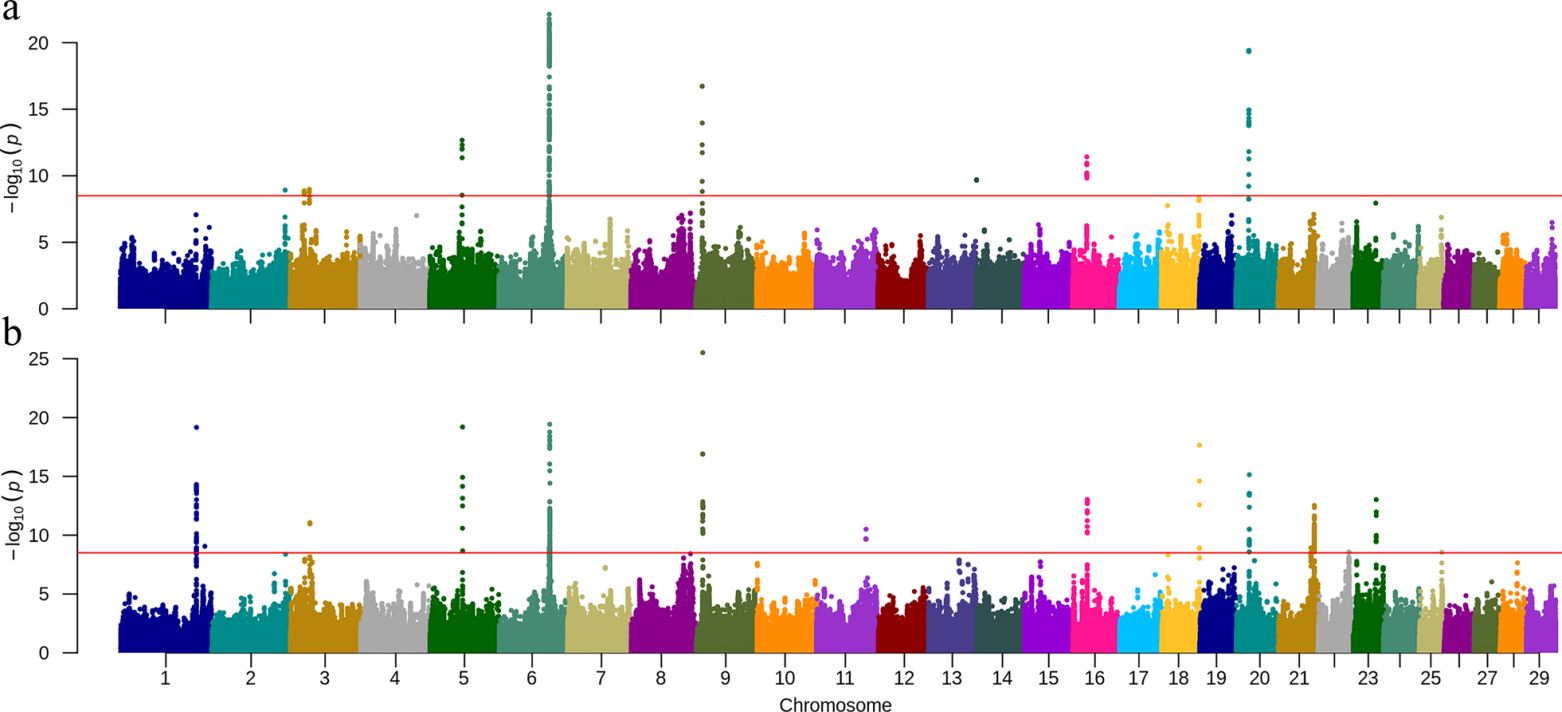
Manhattan plot for multi-trait meta-analysis by MTAG [40]. **a** clinical mastitis; **b** somatic cell score. The red horizontal line indicates the genome-wide significance level [− log10(p) = 8.5]

The multi-trait meta-analysis using MTAG of CM (Fig. 3a) and SCS (Fig. 3b) showed mostly consistent results regarding QTL location for these two mastitis-related traits. One salient difference is that there were two QTL on BTA1 for SCS but none for CM. These two QTL overlapped with results from MR-MEGA_SCS and were located at 131 Mb and 145 Mb (Table 1). However, the lead SNPs suggested by MTAG were different from those highlighted by MR-MEGA_SCS. The lead SNPs on BTA1 for MTAG_SCS were BTA1: 131,508,931 (rs133645774) and BTA1: 145,932,212 (rs469947398). On BTA2, the MTAG_SCS did not identify a genome-wide significant association, but detected one QTL for CM, which was similar to the MR-MEGA_CM result. On BTA3, MTAG detected one new QTL for CM compared to the single-breed GWAS and the meta-analysis for both traits, with the lead SNP located at BTA3: 24,114,904 (rs385025933), which is an intergenic variant near gene *TBX15*. On BTA5, MTAG_CM and MTAG_SCS both identified a novel QTL at 56 Mb, with two different lead SNPs: BTA5: 56,290,204 (rs109848760, MTAG_CM) and BTA5: 56,332,715 (rs208358909, MTAG_SCS). Because of having two lead SNPs, there are two ‘nearest genes’ to this QTL: *LRP1* and *STAT6*. The QTL identified on BTA6, BTA9, BTA16, and BTA20 were consistent between MTAG and MR-MEGA for both traits, with the adjacent of the lead SNPs in each QTL between two traits. On BTA11, MTAG_SCS detected a novel QTL, with BTA11: 85,622,206 (rs382615161) as lead SNP, which is an intergenic variant near TRIB2. On BTA14, the QTL was detected using MTAG_CM but not using MTAG_SCS. On BTA18, BTA21, BTA22, BTA23, and BTA25 QTL were only detected using MTAG_SCS, of which the QTL identified on BTA23 and BTA25 are novel QTL (Table 2). Moreover, the QTL on BTA23 was one of the QTL that was detected in single breed analyses but not in the MR-MEGA_SCS analysis. Compared to all reported QTL for CM and SCS in Animal QTLdb [15], the QTL on BTA3, BTA23, and BTA25 are new QTL.

**Table 2:** Additional novel lead SNPs for QTL identified by multi-trait meta-analysis (MTAG [40]), along with their functional annotation and nearest gene

BTA	BP	QTL	rsid	− log10(p)	Annotation	Nearest gene
3	24,114,904	23,865,017–24,365,480	rs385025933	8.87	Intergenic variant	*TBX15*
5	56,290,204	56,040,322–56,542,938	rs109848760	12.68	Intron variant	*LRP1*
11	85,622,206	85,372,220–85,872,367	rs382615161	10.51	Intergenic variant	*TRIB2*
23	39,530,196	39,295,040–39,780,965	rs136857507	13.02	Intron variant	*KIF13A*
25	38,531,214	37,561,390–38,781,883	rs383719916	8.54	Intron variant	*LOC618542*

The additional lead SNPs discovered by MTAG addition to meta-analysis (MR-MEGA [38]) for both traits

### Validation of candidate CNV in GC for BTA6 QTL variants

In a Dutch Holstein study, Lee et al. identified a CNV which could be the causal variant underlying the BTA6 QTL for mastitis resistance [29]. Our meta-analysis study involved only SNPs and small insertion and deletions (INDELs) and therefore could not test CNVs. We therefore verified whether our top associated SNPs and the reported CNV tag the same QTL. We called structural variant genotypes in WGS data from 567 individuals from Nordic Holstein, Nordic Red, Jersey, and Brown Swiss cattle (see Methods), and identified a 12 kb CNV located at BTA6: 86,949,652–86,961,433. The coordinates of this CNV agree well with those reported by Lee et al., (BTA6: 86,949,651–86,961,428) (location updated by liftover [54]). The CNV was segregating in Nordic Holstein and Nordic Red cattle but was not detected in Jersey cattle. Of note, our study showed that the QTL was identified in Nordic Holstein and Nordic Red cattle, but not in Danish Jerseys.

Nordic Holstein cattle had between two and tenfold more reads mapped to the CNV (see Additional file 2: Figure S6) compared to other regions on BTA6, which was understood as 2 to 10 copies of the 12 kb segment and agrees well with the copy number distribution observed by Lee et al. [29]. The wild type allele (1 copy on each homologous chromosome) was the most abundant, leading to individuals with 2 copies. As individual-level WGS data for the mapping populations were not available, we checked the LD of the CNV tag SNPs from Lee et al. [29] with the CNV we found in the Nordic Holstein animals (Table 3). The SNP BTA6: 86,951,401 (rs110813063) and BTA6:86,956,804 (rs110611635), which were high ranking SNP in MTAG_CM and MTAG_SCS, had perfect LD with the CNV. Three high ranking SNPs for MR-MEGA_CM and MR-MEGA_SCS, BTA6: 86,954,479 (rs109996811), BTA6: 86,954,484 (rs109381427), and BTA6: 86,954,490 (rs110242236) had LD of 0.89 with the CNV. These three SNPs are tagging SNPs for the CNV from the previous study [29]. Moreover, the lead SNP from MR-MEGA_CM, BTA6: 86,940,863 (rs210373936), had an LD of 0.81 with the CNV. The lead SNP for MTAG_CM and MTAG_SCS, BTA6: 86,991,630 (rs436532576) had an LD of 0.9 with the CNV. The two latter SNPs are located up-stream and down-stream, respectively, of the CNV. Moreover, two other SNPs, BTA6: 86,986,115 (rs109893390, a missense deleterious mutation for a novel gene *ENSBTAG00000049290* and an intron variant for *GC*) and BTA6: 87,324,678 (rs110326785, a missense mutation of *NPFFR2*) both had LD larger than 0.80 with the CNV, which indicated that the signal for these three genes, *ENSBTAG00000049290*, *GC,* and *NPFFR2*, could be due to a common causal factor. The Nordic Red cattle showed similar LD patterns between these SNPs and the CNV as for the Nordic Holstein cattle (data not shown).

**Table 3:** Linkage disequilibrium between copy number variants in the *GC* gene and their flanking variants

Rs-ID	Position in Bp		LD (Lee et al. [29])	LD (Nordic Holstein)	Ranking
	UMD3.1	ARS-UCD1.2			
rs210373936	88,672,979	86,940,863	NR	0.81	39/378/1/3/655/727
rs110813063	88,683,517	86,951,401	1	1	38/2/NA/NA/6/3
rs109996811	88,686,597	86,954,479	≥ 0.98	0.89	28/23/2/5/652/773
rs109381427	88,686,600	86,954,484	≥ 0.98	0.89	28/23/2/5/652/773
rs110242236	88,686,606	86,954,490	≥ 0.98	0.89	28/23/2/5652/773
rs110611635	88,688,920	86,956,804	≥ 0.98	1	31/3/NA/NA/2/2
rs436532576	88,723,742	86,991,630	NR	0.90	40/2/NA/NA/1/1
rs108952128	NR	87,000,654	NR	0.66	11/8/8/1/NA/NA

The LD between tagging SNPs for the GC copy number variant (CNV) reported by Lee et al. [29] and the lead SNPs from the current study with the CNV in Nordic Holstein cattle. “Ranking” indicates the ranking of the SNP based on p-value for the following analyses and ordered as: highest ranking in single-trait analysis in any of breed for clinic mastitis (CM) and similarly for somatic cell score (SCS), single-trait meta-analysis of CM, single-trait meta-analysis of SCS, multi-trait analysis of CM and multi-trait analysis of SCS. NR indicates the variant was not reported in the previous study [29]. NA means the variant is not significant in our analyses

### Variant annotation around lead SNPs

The SNPs near the lead SNPs of the QTL are potential causal variants. We annotated all the significant variants within a 1 Mb flanking area of lead SNPs, which resulted in 11 SNPs (Table 4) with potential effects to alter the coding sequence of genes or regulatory elements that could be considered as potential causal variant candidates. On BTA6, we selected two SNPs as putative causal variants, BTA6: 86,986,115 (rs109893390) and BTA6: 87,324,678 (rs110326785). On BTA14, we observed three tolerated missense variants for *CPSF1*, *SLC52A2,* and *DGAT1*. On BTA18, BTA18: 65,188,613 (rs41901751) is an intronic variant of a long non-coding RNA. On BTA19, BTA19: 7,311,199 (rs110542780) is a tolerated missense variant for *ENSBTAG00000038823*, with a *GERP* score of 1.69. On BTA20, we included two intronic variants for *MAP3K1*, both affecting the ncRNA LOC104975241. BTA21: 62,941,833 (rs136844062) is an intronic variant of a non-coding RNA that is close to *VRK1*. Within the QTL region on BTA22, we obtained a strongly significant SNP that was annotated as a tolerated missense variant of the *LTF* gene.

**Table 4:** Candidate causal mutations selected based on annotation of their potential effect on gene products

SNP	Analysis	− log10(p)	Ranking	gene/biotype	Effect (SIFT score)
6: 86,986,115	MTAG_CM, MTAG_SCS	21.02/17.99	14/7	Novel gene *ENSBTAG00000049290*	Missense K15Q, deleterious (0)
6: 87,324,678	MTAG_CM, MTAG_SCS	18.50/10.04	318/496	*NPFFR2*	Missense E406K, tolerated (0.58)
14: 550,784	MR-MEGA_CM	10.49	46	*CPSF1*	Missense T430I, tolerated (0.13)
14: 579,239	MR-MEGA_CM	10.58	22	*SLC52A2*	Missense K242E, tolerated (0.13)
14: 611,019-20	MR-MEGA_CM	10.56	32	*DGAT1*	Missense K232A, tolerated (0.19)
18:65,188,613	MTAG_SCS	17.65	1	Non-coding transcript	lncRNA
19: 7,311,199	MR-MEGA_SCS	9.26	265	Novel gene	Missense V361I, tolerated (0.19)
20: 22,385,791	MR-MEGA_CM, MR-MEGA_SCS	20.14/13.09	2/ 1	*LOC104975241*(*MAP3K1*, intron)	ncRNA
20: 22,386,425	MR-MEGA_CM	21.22	1	*LOC104975241* (*MAP3K1*, intron)	ncRNA, deletion
21: 62,941,833	MR-MEGA_SCS	17.55	1	Non-coding transcript	lncRNA
22: 52,960,814	MR-MEGA_SCS	9.09	150	*LTF* lactotransferrin	Missense I145V, tolerated (0.2)

### Gene-based analysis

To further increase the power of detecting candidate genes, we conducted gene-based association analyses for the meta- and multi-trait analyses for both traits. The gene-based analysis indicated in total 64 genome-wide significant genes (see Additional file 1: Tables S7 and S8). Among these, *STAT6*, *GC*, *TTLL5*, *ATP9A*, *ADCK5*, *ENSBTAG00000050669*, *NOG*, *MAP3K1*, and *LTF* were the nearest genes to lead SNPs. Without a criterion to further prioritize these genes, we only kept the ones with biological support (see Additional file 1: Table S9) for downstream analysis.

Some of the nearest genes had biological support from GO biological process terms “defense response”, “mammary gland epithelial cell proliferation”, “mammary gland morphogenesis”, and “negative regulation of type 2 immune response” (*STAT6*), or “antibacterial humoral response”, “antimicrobial humoral immune response mediated by antimicrobial peptide”, and “defense response to Gram-negative bacterium” (*LTF*). In the MPD [49], abnormal mammary gland morphology, and abnormal immunoglobulin level were reported in mice for *STAT6* mutations and abnormal inflammatory response and abnormal T cell differentiation for *GC*.

Among the genes prioritized based on biological support, some genes are annotated with GO terms “T cell differentiation” (*SOX14*, *BCL11B*), “somatic hypermutation of immunoglobulin genes” (*MCM3AP*), “response to steroid hormone (*PAQR7*)”, “negative regulation of respiratory burst involved in inflammatory response”, “positive regulation of regulatory T cell differentiation”, and “regulation of adaptive immune response” (*DUSP10*), “Immune response” and “inflammatory response” (*CCRL2*), “negative regulation of macrophage cytokine production” (*TGFB3*), “wound healing” (*PLEC*). Furthermore, the MPD [49] suggested relevant phenotypes for genes where mutations in mice affect B cell number or differentiation (*MCM3AP*, *RBM15 TRIB2*, *PICALM*), or T cell number, differentiation and morphology (*GC*, *DUSP10*, *PGAP3*, *SEPTIN9*, *BCL11B*, *DCK*, *SEPTIN9*, *PGAP3*), decreased macrophage proliferation (*NPFFR2*), abnormal immune system physiology (*NFATC2*), mammary gland development (*DGAT1*), increased susceptibility to bacterial infection (*HSF1*), abnormal wound healing (*PLEC*), and abnormal mammary gland growth during pregnancy (*TNFRSF11A*).

### Potential regulation with eQTL

Significant associations between gene expression data (from CattleGTEx [35]) and variants that were identified to be associated with CM and SCS in both single-trait meta-analyses and multi-trait meta-analyses were identified by checking the overlap of significant SNPs. We found only four significant SNPs to have a significant *cis* effect (< 1 Mb) on gene expression. Four variants (rs133257289 in liver, rs137491588 in uterus, rs135442643 in blood, rs135443540 in mammary) that were identified as cis-eQTL for *DGAT1* on BTA14 were also associated with CM (see Additional file 1: Table S10).

Most SNPs that were significant in both the meta-analysis/multi-trait analysis and the eQTL analysis were trans-eQTL (on different chromosome) (see Additional file 3: Table S11). For CM, 45 significant SNPs were also trans-eQTL (false discovery rate, FDR < 0.05) in three different tissues or cells (macrophages, adipose and ovary) for 57 genes from 19 chromosomes. Fifty-two significant SNPs for SCS were associated with trans-eQTL (FDR < 0.05) from eight different tissues or cells (adipose, macrophages, muscle, intramuscular muscle, ovary, liver, milk, uterus) for 61 genes from 23 chromosomes.

**Fig. 4:**
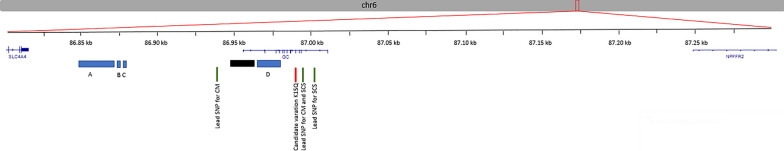
Regional plot of the trans-eQTL located around *GC* CNV on chromosome 6. Integrative Genomics Viewer [74] representation of the trans-eQTL located around GC CNV on chromosome 6. Under the gene track, the black color block indicates the GC CNV. The blue color blocks named A, B, C and D are blocks of SNPs that are significantly associated with either CM or SCS in the current analyses that were also trans-eQTL in CattleGTEx. The green lines show the lead SNPs from the current study and red line shows one of the candidate causal variants

Most of the trans-eQTL SNPs were from BTA6 for both traits and were located as blocks around the multi-allelic CNV region that encompasses the *GC* gene (Fig. 4) that has been associated with mastitis resistance in dairy cattle [29]. The SNP blocks A+C were associated with the expression of *PTGES* (BTA11), while block B affected the expression of *PEX12* (BTA19). The SNPs in block D had trans effects on 43 genes from multiple chromosomes. These genes were enriched in pathways for blood coagulation and plasminogen activating cascade.

### Enrichment of genomic features

We saw an overlap between eQTL and SNPs that were significantly associated with CM or SCS. One strategy to further investigate the effect of regulatory elements on mastitis is an enrichment analysis. To better represent the genomic features across the genome, we collected coordinates for 5ʹ UTR regions, 3ʹ UTR regions, general transcripts, CpG islands, and 13 putative regulatory elements across eight tissues [53]. The putative regulatory elements included active element, active enhancer, active promoter, active transcription start site (TSS), CCCTC-binding factor (CTCF) active TSS, CTCF enhancer, CTCF promoter, flanking TSS, insulator, poised promoter, polycomb repressed, primed enhancer, and promoter. The tissues were adipose, cerebellum, cortex, hypothalamus, liver, lung, muscle, and spleen.

**Fig. 5:**
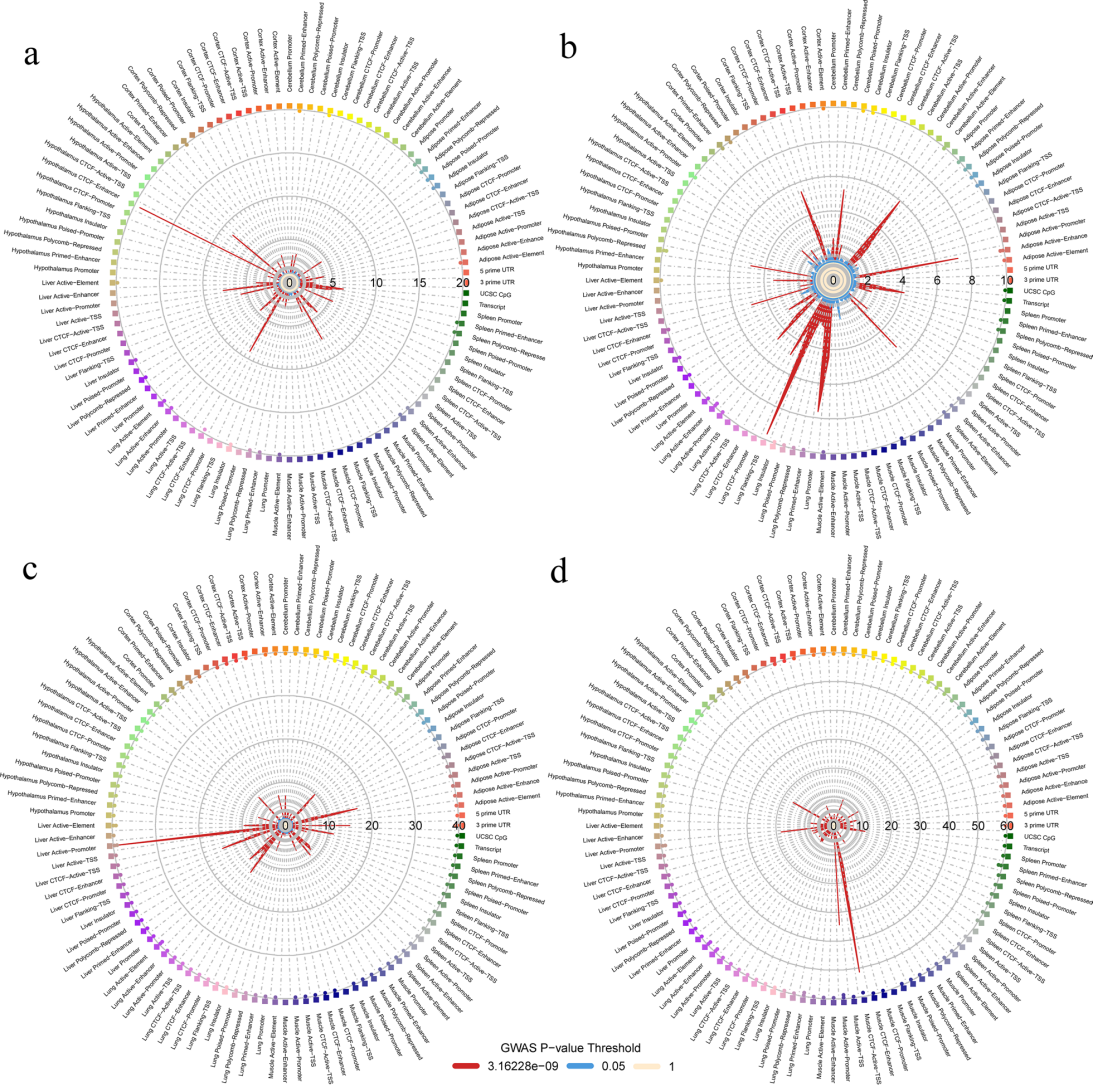
Enrichment analysis. Enrichment of significant variants from the single-trait meta-analysis and multi-trait meta-analysis in genomic features identified from a previous study [53]. The Radial plot shows **a** the enrichment (OR) in meta-analysis of clinical mastitis; **b** OR in meta-analysis of somatic cell score, **c** OR in multi-trait analysis of clinical mastitis, **d** OR in multi-trait analysis of somatic cell score. Squares on the outside of the circle are sorted by tissue for each GWAS significance threshold for 1, 0.1, 0.01, 1^e−04^, 1^e−06^ and 3.16^e−09^ (shown by inner colours and bottom legend)

The summary statistics of MR-MEGA_CM identified CTCF enhancer in lung as a significantly enriched feature for the genome-wide significant variants (Fig. 5a). Three key variants were proposed by the analysis to drive the enrichment, on BTA9, BTA20, and BTA21. For MR-MEGA_SCS, we detected three features that were significantly enriched: active enhancer in adipose, polycomb repressed elements in hypothalamus, and active enhancer in lung (Fig. 5b and Additional file 4: Table S12). The potential key SNPs driving these enrichments are located on BTA9 (rs210770707), BTA10 (rs42248532), BTA13 (rs135899189), BTA19 (rs134402075 for adipose, rs443527170, rs134693405, rs110579341, rs109651074 for hypothalamus, and rs134402075, rs110579341 for lung), BTA20 (rs110323061 for adipose and rs110323061 for lung), and BTA21 (rs134705012 for adipose, rs136844062 for hypothalamus, and rs134705012 for lung). For MTAG_CM, seven enriched features were detected: 3’ UTR, active element in liver, active promoter in liver, and active element in lung (Fig. 5c and Additional file 4: Table S12). The potential key SNPs driving this enrichment were distributed on BTA5 (rs109848760), BTA6 (rs110076968 for 3ʹ UTR, rs110076968, rs436532576, rs436532576 for liver, and rs110076968 for lung), BTA9 (rs109335443 for both liver and lung), BTA16 (rs381913651 for both 3ʹ UTR and lung, and rs379742673 for lung), and BTA20 (rs209103569 for both liver and lung). Lastly, the MTAG_SCS detected six enriched features: active promoter in liver, active element in lung, active enhancer in lung, primed enhancer in lung, CTCF active TSS in muscle, and primed enhancer in spleen (Fig. 5d and Additional file 4: Table S12). The potential key SNPs driving these enrichments were distributed on BTA3 (rs43335461 and rs109100470 for both lung and spleen), BTA5 (rs208358909 for muscle and spleen), BTA6 (rs436532576 for liver), BTA9 (rs134514522 for both elements for lung), BTA11 (rs382615161 for lung), BTA16 (rs379742673 for lung and Spleen and rs381913651 for lung), BTA20 (rs208280837 for liver), and BTA21 (rs110078300 for both elements for lung and Spleen).

### Comparison with GWAS in US Holstein cattle

Comparing our results to a large-scale GWAS in another population can reveal the power of the current study. A previous SCS GWAS using a large population of US Holstein cattle [55] reported results based on the UMD3.1 assembly [56]. We performed a lift over [54] of the marker locations from UMD3.1 to ARS-UCD1.2 to enable comparison with our results. Since the US Holstein study used different data processing and GWAS methods than the current study, we did not include their summary statistics in our meta-analysis. In general, the two Manhattan plots were similar for the chromosomes with shared QTL (see Additional file 2: Figure S7). On BTA6, we identified the QTL near *GC*, while the US study indicated one additional QTL around 50.0 Mb. On BTA13, we identified one QTL located around 56.8 Mb, which was only detected in meta-analysis of SCS. This QTL was close to the QTL identified by US study on BTA13. On BTA14, the common QTL between the current and US study was the QTL located at around 0.6 Mb. However, in our analyses, this QTL was identified for CM. We did not identify any QTL on BTA15 contrary to the US study. The QTL on BTA18 and BTA19 are reported in our study and the US study. Furthermore, our data suggested that both CM and SCS have QTL on BTA18 and BTA19.

### Putative candidate mutations/genes

Based on the single trait meta-analysis, multi-trait analysis, and multiple post-GWAS analysis, we propose 31 putative causal genes (with biological support from GO, Kyoto encyclopedia of genes and genomes, KEGG, or/and MPD) (Table 5). Some of the QTL had more than one putative causal gene. However, we had 11 QTL without putative causal genes: BTA3:24.1 Mb, BTA9:10.5 Mb, BTA11:12.1 Mb, BTA13:56.9 Mb, BTA18:44.4 Mb, BTA19:31.5 Mb, BTA20:39.5 Mb, BTA21:57.1 Mb, BTA23:39.5 Mb, BTA25:38.5 Mb, and BTA29:46.6 Mb. In addition, for some of the putative causal genes, we were able to propose putative causal variants. In total, we propose 14 putative causal variants (Table 5).

**Table 5:** Putative causal genes and variants based on all analyses undertaken

BTA	Gene location	Gene ID	Gene	Statistics	Functional	Variants
1	131,371,915–131,372,637	ENSBTAG00000019535	*SOX14*	Nearest gene	GO: T cell differentiation	NA
1	145,931,228–145,971,979	ENSBTAG00000003148	*MCM3AP*	Nearest gene	MPD: decreased B cell number	NA
2	127,217,390–127,222,938	ENSBTAG00000021787	*PAQR7*	Nearest gene	GO: response to steroid hormone	NA
3	33,088,523–33,095,448	ENSBTAG00000012555	*RBM15*	Nearest gene	MPD: abnormal B cell differentiation	NA
4	10,203,048–10,206,518	ENSBTAG00000051416	*NA*	Nearest gene/Gene analysis	NA	NA
5	56,230,519–56,310,118	ENSBTAG00000010830	*LRP1*	Nearest/ Enrichment analysis	NA	rs109848760
5	56,325,609–56,339,539	ENSBTAG00000006335	*STAT6*	Nearest gene/Gene analysis/trans-eQTL/ Enrichment analysis	Go: defense response, mammary gland epithelial cell proliferation, mammary gland morphogenesis, negative regulation of type 2 immune response; KEGG: Immune system, Immune disease; MPD: abnormal mammary gland morphology, abnormal immunoglobulin level	rs208358909
5	88,262,950–88,412,938	ENSBTAG00000019294	*ABCC9*	Nearest gene/lead SNP	GO: defense response to virus	rs209893772
6	86,319,005–86,345,274	ENSBTAG00000012397	*DCK*	Gene analysis	MPD: increased macrophage cell number, abnormal response to infection	NA
6	86,953,984–87,007,062	ENSBTAG00000013718	*GC*	Nearest gene/Gene analysis	MPD: abnormal inflammatory response, abnormal T cell differentiation	rs436532576
6	86,985,349–86,987,171	ENSBTAG00000049290	Novel gene (*GC* intron)	VEP	NA	rs109893390
6	87,248,937–87,325,253	ENSBTAG00000009070	*NPFFR2*	Gene analysis/VEP	MPD: decreased macrophage proliferation	rs11032678
10	86,918,978–87,207,088	ENSBTAG00000025403	*TTLL5*	Nearest gene/gene analysis	NA	rs42248532
11	85,237,104–85,266,681	ENSBTAG00000016045	*TRIB2*	Nearest gene	MPD: decreased B cell number	NA
13	79,245,983–79,384,839	ENSBTAG00000018270	*NFATC2*	Gene analysis	GO: B cell receptor signaling pathway, positive regulation of B cell proliferation; MPD: abnormal immune system physiology	NA
14	603,813–612,791	ENSBTAG00000026356	*DGAT1*	Gene analysis/VEP/ cis-eQTL	MPD: abnormal mammary gland development	rs109234250
14	613,328–634,349	ENSBTAG00000020751	*HSF1*	Gene analysis	KEGG: Infectious disease: bacterial; MPD: increased susceptibility to bacterial infection	NA
14	839,972–896,647	ENSBTAG00000011922	*PLEC*	Gene analysis	GO: wound healing; MPD: abnormal wound healing, abnormal T cell physiology	NA
14	542,386–556,837	ENSBTAG00000008355	*CPSF1*	Gene analysis/VEP	NA	rs134432442
14	578,057–580,805	ENSBTAG00000000857	*SLC52A2*	Gene analysis/VEP	NA	rs134364612
16	25,186,203–25,227,307	ENSBTAG00000001729	*DUSP10*	Nearest gene/lead SNP	GO: negative regulation of respiratory burst involved in inflammatory response, positive regulation of regulatory T cell differentiation, regulation of adaptive immune response; MPD: abnormal adaptive immunity, increased activated T cell number, decreased T cell proliferation	
18	64,961,803–64,989,519	ENSBTAT00000053442	*LOC100124497*	Nearest gene/VEP	NA	rs41901751
19	7,389,042–7,389,740	ENSBTAG00000040282	*NOG*	Nearest gene/ Gene analysis	GO: wound healing	NA
19	40,047,113–40,061,042	ENSBTAG00000011732	*PGAP3*	Nearest gene/ Gene analysis	MPD: abnormal T cell morphology	NA
19	54,497,005–54,676,832	ENSBTAG00000002633	*SEPTIN9*	Nearest gene/ Gene analysis	KEGG: Infectious disease: bacterial; abnormal T cell differentiation	NA
20	22,314,474–22,323,346	ENSBTAG00000013426	*SETD9*	Gene analysis	NA	NA
20	22,340,163–22,417,428	ENSBTAG00000013790	*MAP3K1*	Nearest gene/Gene analysis/ Enrichment analysis/ Lead SNP/VEP	NA	rs110323061, rs380944374
21	64,193,536–64,290,496	ENSBTAG00000018019	*BCL11B*	Nearest gene	GO: alpha–beta T cell differentiation; MPD: abnormal T cell differentiation	NA
22	52,952,571–52,986,619	ENSBTAG00000001292	*LTF*	Nearest gene/Gene analysis/VEP	GO: antibacterial humoral response, antimicrobial humoral immune response mediated by antimicrobial peptide, defense response to Gram-negative bacterium; KEGG: Exosomal proteins of breast milk	rs109741625
22	52,998,333–53,000,232	ENSBTAG00000006155	*CCRL2*	Nearest gene	GO: Immune response, inflammatory response; MPD: abnormal T-helper 2 physiology	NA
24	60,733,395–60,790,306	ENSBTAG00000007569	*TNFRSF11A*	Gene analysis	GO: mammary gland alveolus development, adaptive immune response; MPD: abnormal mammary gland growth during pregnancy, abnormal negative T cell selection, decreased B cell number	NA
29	9,519,111–9,620,607	ENSBTAG00000001657	*PICALM*	Nearest gene/ trans-eQTL	MPD: abnormal B cell differentiation	NA

## Discussion

### Meta-analysis approach

In this study, single- and multi-trait meta-analyses of several independent GWAS studies increased the power to map genetic variants affecting two related complex traits by leveraging GWAS summary statistics from 8 studies on CM and 14 studies on SCS in six dairy cattle breeds. Combining the summary statistics from multiple breeds for two traits required the application of appropriate methods to account for population and trait differences. Due to strong within breed selection, we expect some QTL to be private to a breed. Such private QTL will be diluted or even disappear in a meta-analysis. We observed a clear advantage of using MR-MEGA over METAL for GWAS meta-analysis in this study. While METAL detected only a well-known locus for CM, MR-MEGA detected 15 association signals. Commercial dairy cattle breeds are under strong genetic selection within breed and a limited number of bulls are used for insemination each year. Therefore, large differences in allele frequencies at the QTL are expected both as a result of artificial selection and genetic drift. The observed increased power in our study for MR-MEGA could be due to its ability to detect heterogeneity in allelic effects between ancestry groups [38], i.e. breeds or populations in the case of this study in dairy cattle.

The accuracy of the marker genotypes is one of the key components of accurate QTL mapping. In this study, each single analysis used the threshold of imputation accuracy R^2^ larger than 0.4 to filter the marker set for GWAS. To minimize the false positive rate while maintaining a large number of variants, we used genotype dosage instead of genotype to perform the GWAS. The genotype dosage is linear transformation of the posterior genotype probabilities and can take the accuracy of imputation into account when performing the GWAS.

Meta-analysis of summary statistics has proven to be a powerful strategy, not only because it can improve the power of detection of alleles with small effect, but it also overcomes obstacles experienced in data handling and sharing (technical, e.g. storage of the raw data, network bandwidth for transferring the raw data, computational resource for running all samples, and data sharing regulations) [57]. Moreover, multi-trait analysis of highly correlated traits can boost power by using information from multiple traits to prioritize common genetic mechanisms [40]. In human GWAS, both these strategies are commonly used to uncover the effect loci for complex traits [58–61]. Such an effort is not frequent in livestock, but there are a few studies, e.g. meta-analysis of cattle stature [31], milk fat and protein percentage [62, 63], and feed efficiency [64], and multi-trait analysis of various production traits [65, 66] and meat quality traits [67]. In this study, we performed the first large-scale meta-analysis for CM and SCS and multi-trait meta-analysis between these two traits to uncover the genetic architecture of mastitis resistance in dairy cattle.

From this study, we observed an increase in power of QTL detection compared to previous GWAS studies about mastitis. Based on MR-MEGA and MTAG analysis, we detected novel QTL for CM and SCS compared with single trait, single breed analysis. We also detected novel QTL compared with previous studies on these traits that are included in the Animal QTLdb [15], including nine new QTL for MR-MEGA_CM, four novel QTL for MR-MEGA_SCS, and three novel QTL from multi-trait meta-analysis. For SCS, even though there have been many association studies performed previously and the list of QTL in the Animal QTLdb [15] is long (1320 QTL, release 49), we still uncovered new QTL.

Although meta-analysis and post-GWAS analyses help to define QTL regions, the problem remains how to discriminate the causative variants from linked polymorphisms. Recent gene editing techniques may offer a way to test the functional effects of candidate variants to further confirm effects at the cellular or animal level. Our list of prioritized variants could serve as a starting list for such functional validation. The most interesting regions in this respect are located on BTA5, BTA6, BTA14, BTA20, and BTA22.

### The putative causal genes and variants

On BTA5, the candidate region between 56.29–56.34 Mb includes the genes *LRP1* and *STAT6*. In addition to rs109848760 and rs208358909 being lead SNPs based on MTAG multi-trait meta-analysis for, respectively, CM and SCS, these two SNPs showed an enriched localization in liver-active elements, and the latter lead SNP, for SCS, showed a trans-eQTL effect in liver on BTA18 (gene *ANKRD27*) in another study [35]. The first lead SNP, rs109848760, is within an intron of the *LRP1* (LDL receptor related protein) gene, which is involved in intracellular signaling, lipid homeostasis, and clearance of apoptotic cells. The second lead SNP, rs208358909, is a synonymous mutation in the *STAT6* gene, which is involved in several central pathways of the immune system and in mammary gland development. It is unlikely that this SNP itself is the causative variant but if may be in LD with a causative SNP in a regulatory feature, potentially having effects on gene expression.

The best (overall) known QTL region for mastitis and somatic cell count is the one on BTA6 surrounding the gene *GC*. We have detected many potential variants that are associated with mastitis resistance in this region (86.8–87.3 Mb), in addition to the CNV that encompasses an enhancer of the *GC* gene. The SNP with highest significance, rs210373936 (single trait meta-analysis for clinical mastitis), is located downstream of the *GC* gene, outside the CNV region. Based on our results, a promising candidate for further validation would be rs436532576 at BTA6: 86,991,630, which was the lead SNP for multi-trait meta-analysis of both CM and SCS and resides within an intron of the *GC* gene. This SNP has also been reported in 3 French breeds to affect udder depth and SCS [28]. Furthermore, it showed up as the key variant to drive the enrichment of liver active elements and promoters [53]. Another interesting SNP for further study is at BTA6: 86,986,115 (rs109893390), which leads to a deleterious variant within a novel candidate mutation within an intron of the *GC* gene. Another candidate in the region to validate is the missense mutation in the nearby *NPFFR2* gene, which was identified by meta-analyses for both single-trait and multi-trait-analysis for CM, was genome-wide significant in the gene-based GWAS analysis and has been found to be associated with decreased macrophage proliferation in mice [49]. Based on currently available information, it is not possible to separate the effects or verify the different candidate variants further due to the strong LD patterns within the region, but it seems plausible that the CNV is not the only causal variant for all the observed effects. The SNP blocks around the CNV-GC region with trans-eQTL effects may be haplotypes due to recent positive selection, as indicated by Lee et al. [29]. Interestingly, the SNPs in the *GC* gene region have trans-effects on expression of several genes on multiple chromosomes in the CattleGTEx [35].

The QTL region on BTA14 overlaps the well-known QTL candidate for milk yield and composition, *DGAT1*, which has also been reported to be associated with mastitis resistance [68, 69]. Moreover, a bivariate association analysis of the QTL at *DGAT1* showed pleiotropic effects on mastitis resistance and milk yield [68]. Pleiotropic effects on mastitis and milk production have also been reported for the BTA6 QTL (*GC* CNV region) in several breeds [23, 28, 70]. In our meta-analysis, pleiotropic effects on additional traits were not studied, as these were not available for current study. Studying the functional effects of the variants suggested by this study in different breeds may reveal the underlying architecture of the pleiotropy and elucidate the background for allelic dynamics.

On BTA20, an interesting region is around the *MAP3K1* gene, with four lead SNPs from single-trait and multi-trait analyses of both CM and SCS between BTA20: 22,385,791 and 22,428,455. The *MAP3K1* gene is a potential candidate gene, as it is part of many signal transduction cascades, including the ERK and JNK kinase pathways, as well as the NF-kappa-B pathway, and the GO annotation indicated that it is involved in the immune system. The variants at BTA20: 22,385,791 (rs110323061) and BTA20: 22,386,425 (rs380944374) are both located in a ncRNA, LOC104975241, within the first intron of the *MAP3K1* gene. The first variant is a SNP and the second one is a deletion. The intron SNP (rs110323061) was the key variant that drove the enrichment of lung-active-element and the upstream SNP BTA20: 22,422,299 (rs209103569) was the key variant to drive the enrichment of liver and lung active elements and liver active promoters.

On BTA22, a missense mutation I145E (tolerated 0.2) in the lactotransferrin gene was found at 52,960,814 bp. The p-value for this SNP was 10^–9.1^, while for the lead SNP at 52,947,790 bp for SCS (MR-MEGA) the p-value was 10^–14.2^. In the gene analysis, *LTF* ranked 2nd (after *LRRC2*) on BTA22 for SCS. LTF is a multifunctional protein with antimicrobial properties that have an important defense role in innate immunity and has been associated with mastitis resistance in humans [71]. Wojdak-Maksymiec et al. [72] showed parity dependent associations between a SNP (rs109623119) within the *LTF* gene and clinical mastitis in cattle. Interestingly, in a study of alternative splicing associated with mastitis [73], one of the *LTF*- isoforms (Lactoferrin_10) was one of the most under-expressed isoforms in the mastitis samples compared to the healthy samples, which suggests that further analysing the expression of this gene may be important for revealing the mechanisms involved in the development of mastitis.

### The limitations of this study and future perspectives

The cattle genome has not been well annotated, which hampers the effort to explore the genetic determinants for complex traits like mastitis. In our findings, the lead SNPs harbour a long list of intergenic variants (Table 1). Although we included the regulator element annotation from eight tissues [53] and large-scale eQTL mapping from farmGTEx [35], we still had limited information to interpret these variants and important regulatory elements could be hidden among. In recent years, key initiatives have been underway to improve annotation of the cattle genome. The Functional Annotation of Animal Genomes (FAANG, https://www.faang.org/), an international consortium since 2015 [36], aims to generate the genomic feature landscape for several livestock species, including the epigenome, chromatin accessibility, and the transcriptome. Meanwhile, the EU BovReg consortium (https://cordis.europa.eu/project/id/815668, www. Bovreg.eu) started in 2019 and aims at functional annotation of active genomic regions in the bovine genome in various tissues that underly phenotypic diversity and plasticity in cattle. With the information that will be generated by these functional studies, we have the potential to better understand the biological connection between identified genetic variants and mastitis resistance and develop models to integrate knowledge on regulatory variation into genomic selection schemes. The other limitation of interpretation of the lead SNPs is lack of direct functional validation. This could be solved by (1) including the putative causal variants from our findings (Table 5) on the panel for routine genotyping and validate the effect in each population; (2) followed-up by designing molecular biological experiments to confirm the causal relationship.

Enrichment analysis one of the methods to provide additional biological meaning to GWAS results, with statistical support. Our enrichment analysis for general genomic features and tissue specific regulatory elements (Fig. 5.) detected several enrichments. However, some limitations of these analyses include: (1) the extensive LD in the cattle genome could lead to spurious enrichments; (2) the enrichment of a specific tissue should not lead to the conclusion of the causality of such tissue to the trait of study, because the tissue collection is broad but not complete and, in addition, regulatory elements overlap among tissues; and (3) regulatory elements are only predicted by bioinformatic analysis without final experimental validation.

In this study, we considered clinical mastitis phenotypes from different countries as the same trait. Measuring clinical mastitis presents several challenges, including the variability in defining the condition, different measurement methods, the presence of subclinical mastitis, and subjective judgments in symptom assessment. Harmonizing phenotype definition for clinical mastitis can improve power of future meta-GWAS. For some countries, cows’ and bulls’ data were analysed separately, although they are from the same population and are related. This violates the assumption of the independent information from individual studies in a meta-analysis.

## Conclusions

In this study, we collected the largest dataset for mastitis traits, CM and SCS, in dairy cattle. In total, GWAS summary statistics based on data from 30,689 animals for CM and 119,438 animals for SCS from six dairy cattle breeds were combined with meta-analysis methodology to account for breed differences and with multi-trait meta-analysis. We identified 58 lead markers that were associated with mastitis incidence, including 16 novel loci compared with previously identified QTL archived at the Animal QTLdb. Meanwhile, we collected multiple sources of annotation information, including predicted regulatory elements and eQTL from multiple tissues, and designed a comprehensive workflow to prioritize the candidate genes and variants. At last, we proposed 31 candidate genes and 14 possible causal variants that affect mastitis resistance. The data collection and methodology for post-GWAS is a unique resource for livestock genetics research. Due to the importance of mastitis for animal welfare, the knowledge obtained from this study will serve as a primary source for cattle mastitis research, cattle breeding, cattle management, and veterinary medicine.

### Supplementary Information

**Additional file 1: Table S1.** Populations imputed to sequence level for somatic cell count (SCS) and clinical mastitis (CM) GWAS. **Table S2.** The population number for meta-analysis for clinic mastitis and somatic cell count. **Table S3.** The significant SNPs for each population. **Table S4.** The Genomic control parameter for meta-analysis for clinic mastitis and somatic cell count. **Table S5.** The SNPs number after quality control for meta-analysis for clinic mastitis and somatic cell count. **Table S6.** The function analysis of nearest gene, NA means the functional annotation is not related to mastitis resistance. **Table S7.** The gene-based analysis for single-trait meta-analysis exceeding the Bonferroni corrected significant threshold (p value < 1.92e-6 i.e. nominal Type-1 error at 0.05 for total number of 26,071 genes). The –log10(p) are from multiple analysis. **Table S8.** The gene-based analysis for multi-trait meta-analysis exceeding the Bonferroni corrected significant threshold (p value < 1.92e-6 i.e. nominal Type-1 error at 0.05 for total number of 26,071 genes). The –log10(p) are from multiple analysis. **Table S9.** The function analysis of significant genes from gene analysis, NA means the functional annotation is not related to mastitis resistance. **Table S10.** The cis-eQTL from cattle GTEx that are significantly associated in our study.

**Additional file 2: Figure S1.** The allele frequency check of each summary statistics. The allele frequency check of each summary statistics with 1000 bull genome project reference population. The x-axis indicated the allele frequency of the reference panel, the y-axis indicated the allele frequency of the same allele in the examined population. The order are: AgVic somatic cell score for Holstein bulls, AgVic somatic cell score for Holstein cows, AgVic somatic cell score for Jersey bulls, AgVic somatic cell score for Jersey cows, AU clinic mastitis for Holstein bulls, AU clinic mastitis for Jersey bulls, LUKE clinic mastitis for Red bulls, AU somatic cell score for Holstein bulls, AU somatic cell score for Jersey bulls, LUKE somatic cell score for Red bulls, ETH clinic mastitis for Brown Swiss cows, ETH clinic mastitis for Brown Swiss bulls, ETH somatic cell score for Brown Swiss cows, ETH somatic cell score for Brown Swiss bulls, INRAE clinic mastitis for Holstein bulls, INRAE clinic mastitis for Montbeliarde bulls, INRAE clinic mastitis for Normande bulls, INRAE somatic cell score for Holstein bulls, INRAE somatic cell score for Montbeliarde bulls, INRAE somatic cell score for Normande bulls, FBN somatic cell score for Holstein bulls, WUR somatic cell score for Holstein bulls. **Figure S2.** Lambda-N plot to reveal issues with population stratification. The orange line indicates the optimal λGC = 1.Dots are all below 1.1 indicated no population with inflation. **Figure S3.** P-Z plot to reveal analytical issues with beta, standard error and P-values. The filtered dataset showing perfect concordance. The order are: AU clinic mastitis for Holstein bulls, AU clinic mastitis for Jersey bulls, LUKE clinic mastitis for Red bulls, ETH clinic mastitis for Brown Swiss cows, ETH clinic mastitis for Brown Swiss bulls, INRAE clinic mastitis for Holstein bulls, INRAE clinic mastitis for Montbeliarde bulls, INRAE clinic mastitis for Normande bulls, AgVic somatic cell score for Holstein bulls, AgVic somatic cell score for Holstein cows, AgVic somatic cell score for Jersey bulls, AgVic somatic cell score for Jersey cows, AU somatic cell score for Holstein bulls, AU somatic cell score for Jersey bulls, LUKE somatic cell score for Red bulls, ETH somatic cell score for Brown Swiss cows, ETH somatic cell score for Brown Swiss bulls,, INRAE somatic cell score for Holstein bulls, INRAE somatic cell score for Montbeliarde bulls, INRAE somatic cell score for Normande bulls, FBN somatic cell score for Holstein bulls, WUR somatic cell score for Holstein bulls. **Figure S4.** Manhattan plot for meta-analysis of clinic mastitis using METAL software. The red horizontal line indicates the genome-wide significance level [− log10(p) = 8.5]. **Figure S5.** Manhattan plot for meta-analysis of somatic cell score using METAL software. The red horizontal line indicates the genome-wide significance level [− log10(p) = 8.5]. **Figure S6.** The copy numbers of the GC copy number variant (CNV) in Nordic Holstein animals (123 bulls). The x-axis is the copy number inferred from the fold-change for the CNV depth relative to other regions of the same chromosome. The y-axis is the number of animals with that copy number. **Figure S7.** Manhattan plot for meta-analysis of somatic cell score (MR-MEGA_SCS) in the current study and US dataset for the selected chromosomes. The red horizontal line indicates the genome-wide significance level [− log_10_(p) = 8.5 and -log_10_(p) = -8.5]. Results from current study are plotted as -log10(p) values in the y-axis, while the US GWAS results as log_10_(p) values.

**Additional file 3: Table S11.** The trans-eQTL from cattle GTEx that are significantly associated in our study.

**Additional file 4: Table S12.** The enrichment of the predicted regulatory elements.

## Data Availability

For the data used in the Nordic GWAS (AU, Denmark and LUKE, Finland), the phenotypes come from the Nordic Cattle Genetic evaluation (NAV, https://nordicebv.info/) and the genotypes come from the cattle breeding company, Viking Genetics (VG, https://www.vikinggenetics.com/). Reasonable requests for research access to this data can be made directly to the NAV and VG. Genotype and phenotype data of Brown Swiss bulls and cows have been provided by Braunvieh Schweiz (https://homepage.braunvieh.ch/), the data could be available from the authors (hubert.pausch@usys.ethz.ch) on reasonable request, and with permission of Braunvieh Schweiz. Australian farmers and DataGene (http://www.datagene.com.au/) are owners and custodians of the Australian phenotype and genotype data used in this study. Reasonable requests for research access to this data can be made via the authors (iona.macleod@agriculture.vic.gov.au) and will require permission from DataGene under a Data Use Agreement. For the data used in the French GWAS (INRAE, Jouy-en-Josas, France), the phenotypes come from the French national genetic evaluation database and most of the cow genotypes come from genomic selection programs managed by Valogene (https://www.eliance.fr/en/le-reseau/valogene). All data belong to French farmers and cannot be disclosed without their explicit permission. The usage of the French data could be available from the authors (marie-pierre.sanchez@inrae.fr and didier.boichard@inrae.fr) on reasonable request, and permission of French farmers. The genotypes and somatic cell count phenotypes for the GWAS performed at FBN are not publicly available, but are available from the authors (kuehn@fbn-dummerstorf.de) on reasonable request, and with permission of FBN.The genotypes and mastitis phenotypes for the GWAS performed at Wageningen University & Research are not publicly available but are available from the authors (aniek.bouwman@wur.nl) on reasonable request, and with permission of Wageningen University & Research. The genome assembly, annotation and location of the transcript used in the current study are available at Ensembl cow genome page (https://www.ensembl.org/Bos_taurus/Info/Index). The CpG islands were retrieved from UCSC genome browser (https://genome.ucsc.edu/). The functional annotation of protein coding genes is available at UniProt (https://www.uniprot.org/). The whole genome sequencing data of 1000 bull genome project is deposited in NCBI BioProjects with accession number: PRJNA431934, PRJNA238491, PRJDB2660, PRJEB18113, PRJEB1829, PRJEB27309, PRJEB28191, PRJEB9343, PRJNA210519, PRJNA210521, PRJNA210523, PRJNA279385, PRJNA294709, PRJNA316122, PRJNA474946, PRJNA477833, PRJNA494431, PRJDA48395, PRJNA431934, and PRJNA238491. The regulatory elements for cattle liver, lung, spleen, skeletal muscle, subcutaneous adipose, cerebellum, brain cortex, and hypothalamus are available at https://farm.cse.ucdavis.edu/~ckern/Nature_Communications_2020/. The eQTL for 24 major tissues is available at CattleGTEx (https://cgtex.roslin.ed.ac.uk/).
