# Supplementary material for: Meta-analysis of six dairy cattle breeds reveals biologically relevant candidate genes for mastitis resistance
Source: Genet Sel Evol. 2024 Jul 15;56:54. doi: 10.1186/s12711-024-00920-8 (PMC11247842; doi:10.1186/s12711-024-00920-8)
Supplement: Supplementary file 1 — Additional file 1: Table S1. Populations imputed to sequence level for somatic cell count (SCS) and clinical mastitis (CM) GWAS. Table S2. The population number for meta-analysis for clinic mastitis and somatic cell count. Table S3. The significant SNPs for each population. Table S4. The Genomic control parameter for meta-analysis for clinic mastitis and somatic cell count. Table S5. The SNPs number after quality control for meta-analysis for clinic mastitis and somatic cell count. Table S6. The function analysis of nearest gene, NA means the functional annotation is not related to mastitis resistance. Table S7. The gene-based analysis for single-trait meta-analysis exceeding the Bonferroni corrected significant threshold (p value < 1.92e-6 i.e. nominal Type-1 error at 0.05 for total number of 26,071 genes). The –log10(p) are from multiple analysis. Table S8. The gene-based analysis for multi-trait meta-analysis exceeding the Bonferroni corrected significant threshold (p value < 1.92e-6 i.e. nominal Type-1 error at 0.05 for total number of 26,071 genes). The –log10(p) are from multiple analysis. Table S9. The function analysis of significant genes from gene analysis, NA means the functional annotation is not related to mastitis resistance. Table S10. The cis-eQTL from cattle GTEx that are significantly associated in our study. [file 12711_2024_920_MOESM1_ESM.docx]

**Meta-analysis of six dairy cattle breeds reveals biologically Relevant candidate genes for mastitis resistance**

Zexi Cai^1*^, Terhi Iso-Touru^2^, Marie-Pierre Sanchez^3^, Naveen Kadri^4^, Aniek C. Bouwman^5^, Praveen Krishna Chitneedi^6^, Iona M. MacLeod^7,8^, Christy J Vander Jagt^7^, Amanda J Chamberlain^7^, Birgit Gredler-Grandl^5^, Mirjam Spengeler^9^, Mogens Sandø Lund^1^, Didier Boichard^3^, Christa Kühn^6,10^, Hubert Pausch^4^, Johanna Vilkki^2#^, Goutam Sahana^1#^

**Table S1. Populations imputed to sequence level for somatic cell count (SCS) and clinical mastitis (CM) GWAS**

| **Partner** | **Breed** | **Cows / Bulls** | **Software(s) used** | | **Reference population**  **(step1; step2)** | **Imputed dataset** | | |
| --- | --- | --- | --- | --- | --- | --- | --- | --- |
|  |  |  | **Step 1 (10/50K->700K)** | **Step 2 (700K->SEQ)** |  | **Animals** | | **Bi-allelic variants** |
| ETH | Brown Swiss and Original Braunvieh | Bulls | Beagle5 | Beagle5 | Step1: 1166 BSW and OB;  Step2: 372BSW and OB | | 10,419 | 27,214,878 |
| ETH | Brown Swiss and Original Braunvieh | Cows | Beagle5 | Beagle5 | Step1: 1166 BSW and OB;  Step2: 372BSW and OB | | 20,185 | 27,214,878 |
| FBN | Holstein | Cows | Beagle 5.0 | Beagle 5.0 | 1000 bulls: 1568 incl. 844 HOL and 144 CHA | | 1,043 | 19,590,361 |
| AU | Holstein | Bulls | Beagle 5.0 and Minimac4 | Beagle 4, Shapeit and Minimac 4 | Step1: 3383 HD from HOL, JER and RDC; Step2: 2685 seq. | | 5,783 | 20,695,475 |
| AU | Jersey | Bulls | Beagle 5.0 and Minimac4 | Beagle 4, Shapeit and Minimac 4 | Step1: 3383 HD from HOL, JER and RDC; Step2: 2685 seq. | | 1,305 | 20,695,475 |
| LUKE | Nordic Red Cattle | Bulls | Beagle 5.0 and Minimac4 | Beagle 4, Shapeit and Minimac 4 | Step1: 3383 HD from HOL, JER and RDC; Step2: 2685 seq. | | 5,153 | 20,695,475 |
| INRAE | Montbeliarde | Bulls | FImpute3 | Minimac4 | Step1: 522 MON bulls; Step2: 1479 seq. from 17 breeds including 63 MON | | 3226 | 25,050,323 |
| INRAE | Normande | Bulls | FImpute3 | Minimac4 | Step1: 546 NOR bulls; Step2: 1479 seq. from 17 breeds including 44 NOR | | 2749 | 25,050,323 |
| INRAE | Holstein | Bulls | FImpute3 | Minimac4 | Step1: 776 HOL bulls; Step2: 700 HOL bull seq. | | 10,292 | 25,050,323 |
| AgVic | Holstein | Bulls | Fimpute3 | Eagle2 &  Minimac3 | 1000 Bulls Run7 (3090 B. taurus) | | 27,466 | 22,361,416 |
| AgVic | Holstein | Cows | Fimpute3 | Eagle2 &  Minimac3 | 1000 Bulls Run7 (3090 B. taurus) | | 83,798 | 22,361,416 |
| AgVic | Jersey | Bulls | Fimpute3 | Eagle2 &  Minimac3 | 1000 Bulls Run7 (3090 B. taurus) | | 4,959 | 22,361,416 |
| AgVic | Jersey | Cows | Fimpute3 | Eagle2 &  Minimac3 | 1000 Bulls Run7 (3090 B. taurus) | | 11,952 | 22,361,416 |

**Table S2. The population number for meta-analysis for clinic mastitis and somatic cell count.**

| **Partner** | **Breed** | **Bulls/cows** | **Population size** | |
| --- | --- | --- | --- | --- |
|  |  |  | **Somatic cell count** | **Clinic mastitis** |
| ETH | Brown Swiss and Original Braunvieh | Bulls | 3,762 | 2,929 |
| ETH | Brown Swiss and Original Braunvieh | Cows | 11,701 | 1,830 |
| WUR | Holstein | Cows | 2,316 | NA |
| FBN | Holstein | Cows | 134 | NA |
| AU | Holstein | Bulls | 5,720 | 5,720 |
| AU | Jersey | Bulls | 1,300 | 1,300 |
| LUKE | Nordic Red Cattle | Bulls | 5,140 | 5,140 |
| INRAE | Montbeliarde | Bulls | 3,114 | 2,723 |
| INRAE | Normande | Bulls | 2,697 | 2,255 |
| INRAE | Holstein | Bulls | 10,066 | 8,792 |
| AgVic | Holstein | Bulls | 6310 | NA |
| AgVic | Holstein | Cows | 55,547 | NA |
| AgVic | Jersey | Bulls | 1473 | NA |
| AgVic | Jersey | Cows | 10,158 | NA |

**Table S3. The significant SNPs for each population.**

| **Partner** | **Breed** | **Bulls/cows** | **Phenotype type** | **Genome-wide significant SNPs** | |
| --- | --- | --- | --- | --- | --- |
|  |  |  |  | **Somatic cell count** | **Clinic mastitis** |
| ETH | Brown Swiss and Original Braunvieh | Bulls | EBV | 0 | 12 |
| ETH | Brown Swiss and Original Braunvieh | Cows | EBV | 122 | 0 |
| WUR | Holstein | Cows | EBV | 0 | NA |
| FBN | Holstein | Cows | Corrected phenotype | 0 | NA |
| AU | Holstein | Bulls | DRP | 419 | 1,227 |
| AU | Jersey | Bulls | DRP | 1,104 | 0 |
| LUKE | Nordic Red Cattle | Bulls | DRP | 19 | 2,268 |
| INRAE | Montbeliarde | Bulls | DYD | 26 | 17 |
| INRAE | Normande | Bulls | DYD | 0 | 0 |
| INRAE | Holstein | Bulls | DYD | 650 | 1,231 |
| AgVic | Holstein | Bulls | DRP | 2 | NA |
| AgVic | Holstein | Cows | DRP | 61 | NA |
| AgVic | Jersey | Bulls | DRP | 147 | NA |
| AgVic | Jersey | Cows | DRP | 200 | NA |

**Table S4. The Genomic control parameter for meta-analysis for clinic mastitis and somatic cell count.**

| **Partner** | **Breed** | **Bulls/cows** | **Genomic control parameter** | |
| --- | --- | --- | --- | --- |
|  |  |  | **Somatic cell count** | **Clinic mastitis** |
| ETH | Brown Swiss and Original Braunvieh | Bulls | 0.969 | 0.989 |
| ETH | Brown Swiss and Original Braunvieh | Cows | 0.949 | 0.970 |
| WUR | Holstein | Cows | 0.984 | NA |
| FBN | Holstein | Cows | 0.996 | NA |
| AU | Holstein | Bulls | 0.673 | 0.710 |
| AU | Jersey | Bulls | 0.490 | 0.532 |
| LUKE | Nordic Red Cattle | Bulls | 0.678 | 0.761 |
| INRAE | Montbeliarde | Bulls | 1.033 | 1.007 |
| INRAE | Normande | Bulls | 0.997 | 0.997 |
| INRAE | Holstein | Bulls | 1.049 | 0.979 |
| AgVic | Holstein | Bulls | 0.975 | NA |
| AgVic | Holstein | Cows | 1.007 | NA |
| AgVic | Jersey | Bulls | 0.989 | NA |
| AgVic | Jersey | Cows | 0.960 | NA |

**Table S5. The SNPs number after quality control for meta-analysis for clinic mastitis and somatic cell count.**

| **Partner** | **Breed** | **Bulls/cows** | **Number of SNPs considered for association testing** | |
| --- | --- | --- | --- | --- |
|  |  |  | **Somatic cell count** | **Clinic mastitis** |
| ETH | Brown Swiss and Original Braunvieh | Bulls | 16,825,799 | 16,817,149 |
| ETH | Brown Swiss and Original Braunvieh | Cows | 16,750,071 | 16,663,959 |
| WUR | Holstein | Cows | 15,685,889 | NA |
| FBN | Holstein | Cows | 11,132,286 | NA |
| AU | Holstein | Bulls | 15,995,796 | 15,995,796 |
| AU | Jersey | Bulls | 12,569,222 | 12,569,209 |
| LUKE | Nordic Red Cattle | Bulls | 17,134,891 | 17,134,891 |
| INRAE | Montbeliarde | Bulls | 12,683,946 | 12,622,010 |
| INRAE | Normande | Bulls | 12,064,887 | 11,998,527 |
| INRAE | Holstein | Bulls | 12,318,104 | 12,272,960 |
| AgVic | Holstein | Bulls | 14,599,556 | NA |
| AgVic | Holstein | Cows | 14,514,155 | NA |
| AgVic | Jersey | Bulls | 13,035,514 | NA |
| AgVic | Jersey | Cows | 12,564,772 | NA |

**Table S6. The function analysis of nearest gene, NA means the functional annotation is not related to mastitis resistance.**

| **BTA** | **gene** | **loc** | **GO** | **KEGG** | **MPD** |
| --- | --- | --- | --- | --- | --- |
| 1 | *SOX14* | 131,371,915-131,372,637 | T cell differentiation | NA | NA |
|  | *AGPAT3* | 143,941,826-144,025,795 | NA | NA | NA |
|  | *MCM3AP* | 145,931,228-145,971,979 | somatic hypermutation of immunoglobulin genes | NA | decreased B cell number |
| 2 | *TRIM63* | 127,020,869-127,035,929 | NA | NA | NA |
|  | *PAQR7* | 127,217,390-127,222,938 | response to steroid hormone | NA | NA |
| 3 | *CYM* | 32,944,105-32,954,911 | NA | NA | NA |
|  | *TBX15* | 24,129,991-24,254,368 | NA | NA | NA |
|  | *PROK1* | 32,969,972-32,974,969 | NA | NA | NA |
|  | *RBM15* | 33,088,523-33,095,448 | NA | NA | abnormal B cell differentiation |
| 4 | *ENSBTAG00000051416* | 10,203,048-10,206,518 | NA | NA | NA |
| 5 | *ABCC9* | 88,262,950-88,412,938 | defense response to viruses |  | NA |
|  | *LRP1* | 56,230,519-56,310,118 | NA | NA | NA |
|  | *STAT6* | 56,325,609-56,339,539 | defense response, mammary gland epithelial cell proliferation, mammary gland morphogenesis, negative regulation of type 2 immune response | Immune system, Immune disease | abnormal mammary gland morphology, abnormal immunoglobulin level |
| 6 | *GC* | 86,953,984-87,007,062 | NA | NA | abnormal inflammatory response, abnormal T cell differentiation |
| 9 | *bta-mir-30f* | 10,494,819-10,494,902 | NA | NA | NA |
|  | *OGFRL1* | 10,397,908-10,413,050 | NA | NA | NA |
|  | *ENSBTAG00000048046* | 10,632,692-10,633,051 | NA | NA | NA |
| 10 | *TTLL5* | 86,918,978-87,207,088 | NA | NA | NA |
| 11 | *EXOC6B* | 11,625,730-12,344,742 | NA | NA | NA |
|  | *TRIB2* | 85,237,104-85,266,681 | NA | NA | decreased B cell number |
| 13 | *PHACTR3* | 56,695,468-56,901,813 | NA | NA | NA |
|  | *ATP9A* | 79,436,295-79,557,537 | NA | NA | NA |
| 14 | *ADCK5* | 523,820-525,625 | NA | NA | NA |
|  | *ENSBTAG00000053637* | 556,020-570,498 | NA | NA | NA |
| 16 | *DUSP10* | 25,186,203-25,227,307 | negative regulation of respiratory burst involved in inflammatory response, positive regulation of regulatory T cell differentiation, regulation of adaptive immune response | NA | abnormal adaptive immunity, increased activated T cell number, decreased T cell proliferation |
| 18 | *LOC100124497* | 64,948,170-65,182,761 | NA | NA | NA |
|  | *ENSBTAG00000049393* | 43,728,249-43,729,310 | NA | NA | NA |
|  | *ENSBTAG00000050669* | 44,285,683-44,392,228 | NA | NA | NA |
| 19 | *NOG* | 7,389,042-7,389,740 | wound healing | NA | NA |
|  | *ENSBTAG00000052541* | 31,397,197-31,397,490 | NA | NA | NA |
|  | *PGAP3* | 40,047,113-40,061,042 | NA | NA | abnormal T cell morphology |
|  | *SEPTIN9* | 54,497,005-54,676,832 | NA | Infectious disease: bacterial | abnormal T cell differentiation |
|  | *LLGL2* | 55,984,816-56,020,314 | NA | NA | NA |
| 20 | *MAP3K1* | 22,340,163-22,417,428 | NA | NA | NA |
|  | *RAI14* | 39,314,838-39,473,962 | NA | NA | NA |
| 21 | *SLC24A4* | 57,008,449-57,197,392 | NA | NA | NA |
|  | *5S_rRNA* | 55,349,041-55,349,123 | NA | NA | NA |
|  | *BCL11B* | 64,193,536-64,290,496 | alpha-beta T cell differentiation | NA | abnormal T cell differentiation |
| 22 | *LTF* | 52,952,571-52,986,619 | antibacterial humoral response, antimicrobial humoral immune response mediated by antimicrobial peptide, defense response to Gram-negative bacterium | Exosomal proteins of breast milk | NA |
|  | *CCRL2* | 52,998,333-53,000,232 | Immune response, inflammatory response | NA | abnormal T-helper 2 physiology |
| 23 | *KIF13A* | 39,526,102-39,728,514 | NA | NA | NA |
| 24 | *ZCCHC2* | 60,903,462-60,958,117 | NA | NA | NA |
| 25 | *LOC618542* | 38,526,071-38,531,691 | NA | NA | NA |
| 29 | *PICALM* | 9,519,111-9,620,607 | NA | NA | abnormal B cell differentiation |
|  | *ENSBTAG00000050252* | 46,603,761-46,624,255 | NA | NA | NA |

**Table S7. The gene-based analysis for single-trait meta-analysis exceeding the Bonferroni corrected significant threshold (p value < 1.92e-6 i.e. nominal Type-1 error at 0.05 for total number of 26,071 genes). The –log10(p) are from multiple analysis.**

| **BTA** | **GENE ID** | **GENE** | **Location** | **Ranking within QTL (− log10(p))** | | |
| --- | --- | --- | --- | --- | --- | --- |
|  |  |  |  | **MR-MEGA_CM** | **MR-MEGA_SCS** | |
| 4 | *ENSBTAG00000018363* | *RBM48* | 9870503-9877234 | NA | 2 (7.20) | |
| 4 | *ENSBTAG00000051416* | NA | 10203048-10206518 | 1 (7.45) | 1 (14.43) | |
| 6 | *ENSBTAG00000011952* | *SULT1E1* | 85309030-85365114 | 9 (6.43) | NA | |
| 6 | *ENSBTAG00000004793* | *AMBN* | 85968067-85979382 | 8 (8.38) | NA | |
| 6 | *ENSBTAG00000051236* | NA | 86229230-86230375 | 5 (9.88) | 4 (6.08) | |
| 6 | *ENSBTAG00000016290* | *MOB1B* | 86250301-86308361 | 7 (8.98) | NA | |
| 6 | *ENSBTAG00000012397* | *DCK* | 86319005-86345274 | 3 (12.09) | NA | |
| 6 | *ENSBTAG00000002348* | *SLC4A4* | 86381836-86809131 | 2 (16.11) | 2 (12.40) | |
| 6 | *ENSBTAG00000013718* | *GC* | 86953984-87007062 | 1 (24.54) | 1 (17.25) | |
| 6 | *ENSBTAG00000009070* | *NPFFR2* | 87248937-87325253 | 6 (9.41) | 3 (9.29) | |
| 6 | *ENSBTAG00000004921* | NA | 91186036-91199159 | 4 (10.04) | NA | |
| 10 | *ENSBTAG00000025403* | *TTLL5* | 86918978-87207088 | NA | 1 (12.80) | |
| 10 | *ENSBTAG00000012004* | *TGFB3* | 87237844-87271480 | NA | 2 (10.63) | |
| 13 | *ENSBTAG00000018270* | *NFATC2* | 79245983-79384839 | NA | 1 (12.34) | |
| 13 | *ENSBTAG00000007962* | *ATP9A* | 79436295-79557537 | NA | 2 (11.56) | |
| 14 | *ENSBTAG00000007186* | *ARHGAP39* | 359395-414811 | 18 (7.19) | NA | |
| 14 | *ENSBTAG00000046031* | *C14H8orf82* | 416888-419537 | 21 (6.52) | NA | |
| 14 | *ENSBTAG00000004970* | *LRRC24* | 417616-424777 | 20 (6.87) | NA | |
| 14 | *ENSBTAG00000004969* | *LRRC14* | 424798-428272 | 22 (6.47) | NA | |
| 14 | *ENSBTAG00000010276* | *RECQL4* | 428460-434942 | 13 (8.23) | NA | |
| 14 | *ENSBTAG00000007838* | NA | 435003-437076 | 17 (7.25) | NA | |
| 14 | *ENSBTAG00000007835* | *GPT* | 438530-442163 | 19 (7.16) | NA | |
| 14 | *ENSBTAG00000007834* | *PPP1R16A* | 443246-448420 | 16 (7.69) | NA | |
| 14 | *ENSBTAG00000046026* | *SLC39A4* | 533918-538406 | 8 (9.65) | NA | |
| 14 | *ENSBTAG00000008355* | *CPSF1* | 542386-556837 | 1 (11.67) | NA | |
| 14 | *ENSBTAG00000011064* | *ADCK5* | 556020-570498 | 2 (11.50) | NA | |
| 14 | *ENSBTAG00000000857* | *SLC52A2* | 578057-580805 | 4 (10.64) | NA | |
| 14 | *ENSBTAG00000035158* | *TMEM249* | 584956-586597 | 5 (10.60) | NA | |
| 14 | *ENSBTAG00000050112* | *SCRT1* | 594540-600190 | 6 (10.52) | NA | |
| 14 | *ENSBTAG00000026356* | *DGAT1* | 603813-612791 | 3 (11.39) | NA | |
| 14 | *ENSBTAG00000020751* | *HSF1* | 613328-634349 | 9 (9.38) | NA | |
| 14 | *ENSBTAG00000017281* | *OPLAH* | 765421-774581 | 11 (8.56) | NA | |
| 14 | *ENSBTAG00000015040* | NA | 774643-776724 | 15 (7.76) | NA | |
| 14 | *ENSBTAG00000026350* | *SPATC1* | 778853-806391 | 7 (10.17) | NA | |
| 14 | *ENSBTAG00000000312* | *GRINA* | 826740-830066 | 12 (8.34) | NA | |
| 14 | *ENSBTAG00000009677* | *PARP10* | 832766-839743 | 10 (8.86) | NA | |
| 14 | *ENSBTAG00000011922* | *PLEC* | 839972-896647 | 14 (7.83) | NA | |
| 18 | *ENSBTAG00000050669* | NA | 44285683-44392228 | NA | 1 (8.37) | |
| 19 | *ENSBTAG00000021292* | *ANKFN1* | 7149813-7279813 | 1 (8.29) | 1 (11.4) | |
| 19 | *ENSBTAG00000038823* | NA | 7286704-7311297 | 3 (7.80) | 4 (9.21) | |
| 19 | *ENSBTAG00000040282* | *NOG* | 7389042-7389740 | NA | 6 (8.37) | |
| 19 | *ENSBTAG00000008366* | *STAC2* | 39703116-39716321 | NA | 5 (8.82) | |
| 19 | *ENSBTAG00000011732* | *PGAP3* | 40047113-40061042 | NA | 3 (9.61) | |
| 19 | *ENSBTAG00000021468* | *MED24* | 40334163-40360183 | NA | 8 (7.78) | |
| 19 | *ENSBTAG00000045067* | *SNORD124* | 40341515-40341618 | NA | 9 (7.37) | |
| 19 | *ENSBTAG00000012178* | *NR1D1* | 40389125-40397513 | NA | 10 (6.17) | |
| 19 | *ENSBTAG00000002633* | *SEPTIN9* | 54497005-54676832 | NA | 2 (10.91) | |
| 19 | *ENSBTAG00000020067* | *LLGL2* | 55984816-56020314 | 2 (7.88) | NA | |
| 19 | *ENSBTAG00000048685* | NA | 59544583-59551858 | NA | 7 (7.94) | |
| 20 | *ENSBTAG00000013426* | *SETD9* | 22314474-22323346 | 1 (16.00) | 1 (9.11) | |
| 20 | *ENSBTAG00000013790* | *MAP3K1* | 22340163-22417428 | 2 (14.26) | NA | |
| 20 | *ENSBTAG00000010423* | *LIFR* | 35840757-35949859 | NA | 2 (7.49) | |
| 20 | *ENSBTAG00000052650* | NA | 35949939-35952005 | 3 (7.08) | NA | |
| 21 | *ENSBTAG00000006620* | *SLC24A4* | 57008449-57197392 | 1 (12.75) | NA | |
| 21 | *ENSBTAG00000044369* | *bta-mir-2284f* | 57156230-57156291 | 2 (11.03) | 1 (8.62) | |
| 22 | *ENSBTAG00000008013* | *LRRC2* | 52863612-52921087 | NA | 1 (11.51) |  |
| 22 | *ENSBTAG00000018767* | *RTP3* | 52927961-52930665 | NA | 3 (9.23) |  |
| 22 | *ENSBTAG00000001292* | *LTF* | 52952571-52986619 | NA | 2 (11.42) |  |
| 24 | *ENSBTAG00000007569* | *TNFRSF11A* | 60733395-60790306 | NA | 1 (10.63) |  |
| 29 | *ENSBTAG00000000103* | NA | 49542736-49549261 | NA | 1 (6.49) | NA |

**Table S8. The gene-based analysis for multi-trait meta-analysis exceeding the Bonferroni corrected significant threshold (p value < 1.92e-6 i.e. nominal Type-1 error at 0.05 for total number of 26,071 genes). The –log10(p) are from multiple analysis.**

| **BTA** | **GENE ID** | **GENE** | **Location** | **Ranking within QTL (− log10(p))** | |
| --- | --- | --- | --- | --- | --- |
|  |  |  |  | **MTAG_CM** | **MTAG_SCS** |
| 5 | *ENSBTAG00000006324* | *NAB2* | 56340377-56346527 | 1 (11.42) | 2 (15.00) |
| 5 | *ENSBTAG00000006335* | *STAT6* | 56325609-56339539 | 2 (8.59) | 1 (19.30) |
| 6 | *ENSBTAG00000009070* | *NPFFR2* | 87248937-87325253 | 1 (16.10) | NA |
| 18 | *ENSBTAG00000038088* | NA | 65252212-65254845 | 1 (6.26) | 1 (14.69) |
| 25 | *ENSBTAG00000040014* | NA | 38526071-38531691 | NA | 1 (8.44) |

**Table S9. The function analysis of significant genes from gene analysis, NA means the functional annotation is not related to mastitis resistance.**

| **BTA** | **GENE ID** | **GENE** | **GO** | **KEGG** | **MPD** |
| --- | --- | --- | --- | --- | --- |
| 4 | *ENSBTAG00000051416* | NA | NA | NA | NA |
| 4 | *ENSBTAG00000018363* | *RBM48* | NA | NA | NA |
| 5 | *ENSBTAG00000006324* | *NAB2* | NA | NA | NA |
| 5 | *ENSBTAG00000006335* | *STAT6* | defense response, mammary gland epithelial cell proliferation, mammary gland morphogenesis, negative regulation of type 2 immune response | Immune system, Immune disease | abnormal mammary gland morphology, abnormal immunoglobulin level |
| 6 | *ENSBTAG00000013718* | *GC* | NA | NA | abnormal inflammatory response, abnormal T cell differentiation |
| 6 | *ENSBTAG00000002348* | *SLC4A4* | NA | NA | NA |
| 6 | *ENSBTAG00000012397* | *DCK* | NA | NA | increased macrophage cell number, abnormal response to infection |
| 6 | *ENSBTAG00000004921* | NA | NA | NA | NA |
| 6 | *ENSBTAG00000051236* | NA | NA | NA | NA |
| 6 | *ENSBTAG00000009070* | *NPFFR2* | NA | NA | decreased macrophage proliferation |
| 6 | *ENSBTAG00000016290* | *MOB1B* | NA | NA | NA |
| 6 | *ENSBTAG00000004793* | *AMBN* | NA | NA | NA |
| 6 | *ENSBTAG00000011952* | *SULT1E1* | NA | NA | NA |
| 10 | *ENSBTAG00000025403* | *TTLL5* | NA | NA | NA |
| 10 | *ENSBTAG00000012004* | *TGFB3* | negative regulation of macrophage cytokine production | NA | NA |
| 13 | *ENSBTAG00000018270* | *NFATC2* | B cell receptor signaling pathway, positive regulation of B cell proliferation | NA | abnormal immune system physiology |
| 13 | *ENSBTAG00000007962* | *ATP9A* | NA | NA | NA |
| 14 | *ENSBTAG00000008355* | *CPSF1* | NA | NA | NA |
| 14 | *ENSBTAG00000011064* | *ADCK5* | NA | NA | NA |
| 14 | *ENSBTAG00000026356* | *DGAT1* | NA | NA | abnormal mammary gland development |
| 14 | *ENSBTAG00000000857* | *SLC52A2* | NA | NA | NA |
| 14 | *ENSBTAG00000035158* | *TMEM249* | NA | NA | NA |
| 14 | *ENSBTAG00000050112* | *SCRT1* | NA | NA | NA |
| 14 | *ENSBTAG00000026350* | *SPATC1* | NA | NA | NA |
| 14 | *ENSBTAG00000046026* | *SLC39A4* | NA | NA | NA |
| 14 | *ENSBTAG00000020751* | *HSF1* | NA | Infectious disease: bacterial | increased susceptibility to bacterial infection |
| 14 | *ENSBTAG00000009677* | *PARP10* | NA | NA | NA |
| 14 | *ENSBTAG00000017281* | *OPLAH* | NA | NA | NA |
| 14 | *ENSBTAG00000000312* | *GRINA* | NA | NA | NA |
| 14 | *ENSBTAG00000010276* | *RECQL4* | NA | NA | NA |
| 14 | *ENSBTAG00000011922* | *PLEC* | wound healing | MR-MEGA_CM | abnormal wound healing, abnormal T cell physiology |
| 14 | *ENSBTAG00000015040* | *SMPD5* | NA | NA | NA |
| 14 | *ENSBTAG00000007834* | *PPP1R16A* | NA | NA | NA |
| 14 | *ENSBTAG00000007838* | *MFSD3* | NA | NA | NA |
| 14 | *ENSBTAG00000007186* | *ARHGAP39* | NA | NA | NA |
| 14 | *ENSBTAG00000007835* | *GPT* | NA | NA | NA |
| 14 | *ENSBTAG00000004970* | *LRRC24* | NA | NA | NA |
| 14 | *ENSBTAG00000046031* | *C14H8orf82* | NA | NA | NA |
| 14 | *ENSBTAG00000004969* | *LRRC14* | NA | NA | NA |
| 18 | *ENSBTAG00000050669* | NA | NA | NA | NA |
| 18 | *ENSBTAG00000038088* | NA | NA | NA | NA |
| 19 | *ENSBTAG00000021292* | *ANKFN1* | NA | NA | NA |
| 19 | *ENSBTAG00000020067* | *LLGL2* | NA | NA | NA |
| 19 | *ENSBTAG00000038823* | NA | NA | NA | NA |
| 19 | *ENSBTAG00000002633* | *SEPTIN9* | NA | Infectious disease: bacterial | abnormal T cell differentiation |
| 19 | *ENSBTAG00000011732* | *PGAP3* | NA | NA | abnormal T cell morphology |
| 19 | *ENSBTAG00000008366* | *STAC2* | NA | NA | NA |
| 19 | *ENSBTAG00000040282* | *NOG* | wound healing | MR-MEGA_SCS | NA |
| 19 | *ENSBTAG00000048685* | NA | NA | NA | NA |
| 19 | *ENSBTAG00000021468* | *MED24* | NA | NA | NA |
| 19 | *ENSBTAG00000045067* | *SNORD124* | NA | NA | NA |
| 19 | *ENSBTAG00000012178* | *NR1D1* | NA | NA | NA |
| 20 | *ENSBTAG00000013426* | *SETD9* | NA | NA | NA |
| 20 | *ENSBTAG00000013790* | *MAP3K1* | NA | NA | NA |
| 20 | *ENSBTAG00000052650* | NA | NA | NA | NA |
| 20 | *ENSBTAG00000010423* | *LIFR* | NA | NA | NA |
| 21 | *ENSBTAG00000006620* | *SLC24A4* | NA | NA | NA |
| 21 | *ENSBTAG00000044369* | *bta-mir-2284f* | NA | NA | NA |
| 22 | *ENSBTAG00000008013* | *LRRC2* | NA | NA | NA |
| 22 | *ENSBTAG00000001292* | *LTF* | defense response to Gram-negative bacterium, innate immune response in mucosa | NA | NA |
| 22 | *ENSBTAG00000018767* | *RTP3* | NA | NA | NA |
| 24 | *ENSBTAG00000007569* | *TNFRSF11A* | adaptive immune response | NA | abnormal mammary gland growth during pregnancy, abnormal negative T cell selectio, decreased B cell number |
| 25 | *ENSBTAG00000040014* | *LOC618542* | NA | NA | NA |
| 29 | *ENSBTAG00000000103* | *MRPL23* | NA | NA | NA |

**Table S10. The cis-eQTL from cattle GTEx that are significantly associated in our study.**

| **Tissue** | **SNP** | **chr** | **bp** | **Gene** | **P value** | **Gene** | **analysis** |
| --- | --- | --- | --- | --- | --- | --- | --- |
| Liver | 14:544162 | 14 | 544162 | *ENSBTAG00000026356* | 5.97E-16 | *DGAT1* | CM-meta_analysis |
| Uterus | 14:550081 | 14 | 550081 | *ENSBTAG00000026356* | 1.57E-08 | *DGAT1* | CM-meta_analysis |
| Blood | 14:553910 | 14 | 553910 | *ENSBTAG00000026356* | 5.00E-13 | *DGAT1* | CM-meta_analysis |
| Mammary | 14:568472 | 14 | 568472 | *ENSBTAG00000026356* | 2.64E-12 | *DGAT1* | CM-meta_analysis |
