# Supplementary material for: Meta-analysis of six dairy cattle breeds reveals biologically relevant candidate genes for mastitis resistance
Source: Genet Sel Evol. 2024 Jul 15;56:54. doi: 10.1186/s12711-024-00920-8 (PMC11247842; doi:10.1186/s12711-024-00920-8)
Supplement: Supplementary file 2 — Additional file 2: Figure S1. The allele frequency check of each summary statistics. The allele frequency check of each summary statistics with 1000 bull genome project reference population. The x-axis indicated the allele frequency of the reference panel, the y-axis indicated the allele frequency of the same allele in the examined population. The order are: AgVic somatic cell score for Holstein bulls, AgVic somatic cell score for Holstein cows, AgVic somatic cell score for Jersey bulls, AgVic somatic cell score for Jersey cows, AU clinic mastitis for Holstein bulls, AU clinic mastitis for Jersey bulls, LUKE clinic mastitis for Red bulls, AU somatic cell score for Holstein bulls, AU somatic cell score for Jersey bulls, LUKE somatic cell score for Red bulls, ETH clinic mastitis for Brown Swiss cows, ETH clinic mastitis for Brown Swiss bulls, ETH somatic cell score for Brown Swiss cows, ETH somatic cell score for Brown Swiss bulls, INRAE clinic mastitis for Holstein bulls, INRAE clinic mastitis for Montbeliarde bulls, INRAE clinic mastitis for Normande bulls, INRAE somatic cell score for Holstein bulls, INRAE somatic cell score for Montbeliarde bulls, INRAE somatic cell score for Normande bulls, FBN somatic cell score for Holstein bulls, WUR somatic cell score for Holstein bulls. Figure S2. Lambda-N plot to reveal issues with population stratification. The orange line indicates the optimal λGC = 1.Dots are all below 1.1 indicated no population with inflation. Figure S3. P-Z plot to reveal analytical issues with beta, standard error and P-values. The filtered dataset showing perfect concordance. The order are: AU clinic mastitis for Holstein bulls, AU clinic mastitis for Jersey bulls, LUKE clinic mastitis for Red bulls, ETH clinic mastitis for Brown Swiss cows, ETH clinic mastitis for Brown Swiss bulls, INRAE clinic mastitis for Holstein bulls, INRAE clinic mastitis for Montbeliarde bulls, INRAE clinic mastitis for Normande bulls, AgVic somatic cell score for Holstein [file 12711_2024_920_MOESM2_ESM.docx]

**Meta-analysis of six dairy cattle breeds reveals biologically relevant candidate genes for mastitis resistance**

Zexi Cai^1*^, Terhi Iso-Touru^2^, Marie-Pierre Sanchez^3^, Naveen Kadri^4^, Aniek C. Bouwman^5^, Praveen Krishna Chitneedi^6^, Iona M. MacLeod^7,8^, Christy J Vander Jagt^7^, Amanda J Chamberlain^7^, Birgit Gredler-Grandl^5^, Mirjam Spengeler^9^, Mogens Sandø Lund^1^, Didier Boichard^3^, Christa Kühn^6,10^, Hubert Pausch^4^, Johanna Vilkki^2#^, Goutam Sahana^1#^


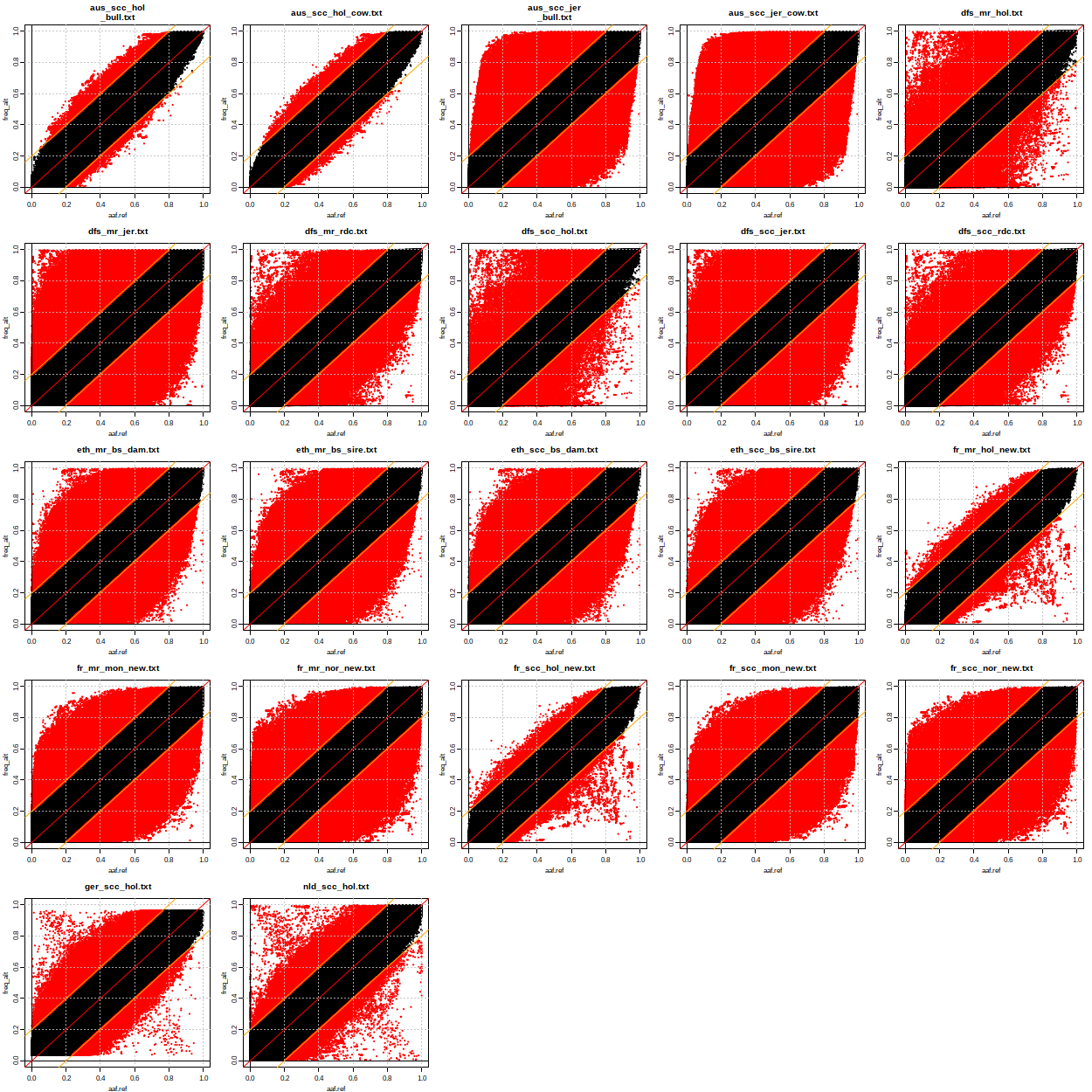


**Figure S1. The allele frequency check of each summary statistics.** The allele frequency check of each summary statistics with 1000 bull genome project reference population. The x-axis indicated the allele frequency of the reference panel, the y-axis indicated the allele frequency of the same allele in the examined population. The order are: AgVic somatic cell score for Holstein bulls, AgVic somatic cell score for Holstein cows, AgVic somatic cell score for Jersey bulls, AgVic somatic cell score for Jersey cows, AU clinic mastitis for Holstein bulls, AU clinic mastitis for Jersey bulls, LUKE clinic mastitis for Red bulls, AU somatic cell score for Holstein bulls, AU somatic cell score for Jersey bulls, LUKE somatic cell score for Red bulls, ETH clinic mastitis for Brown Swiss cows, ETH clinic mastitis for Brown Swiss bulls, ETH somatic cell score for Brown Swiss cows, ETH somatic cell score for Brown Swiss bulls, INRAE clinic mastitis for Holstein bulls, INRAE clinic mastitis for Montbeliarde bulls, INRAE clinic mastitis for Normande bulls, INRAE somatic cell score for Holstein bulls, INRAE somatic cell score for Montbeliarde bulls, INRAE somatic cell score for Normande bulls, FBN somatic cell score for Holstein bulls, WUR somatic cell score for Holstein bulls.


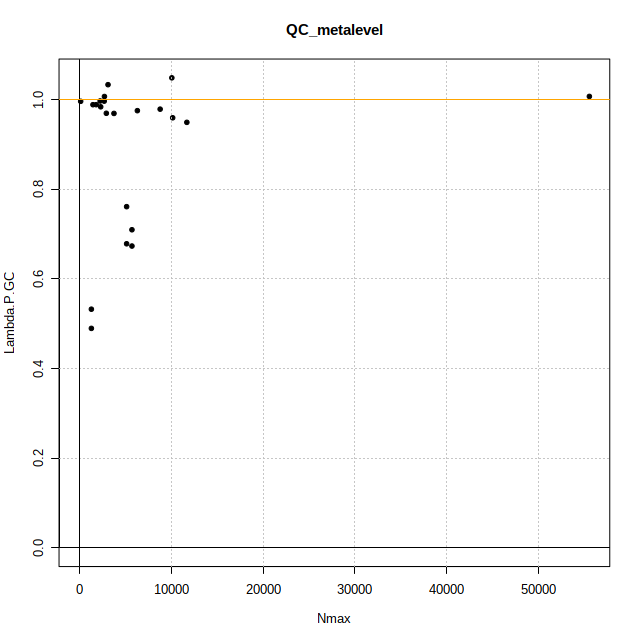


**Figure S2.** **Lambda-N plot to reveal issues with population stratification.** The orange line indicates the optimal λGC=1.Dots are all below 1.1 indicated no population with inflation.


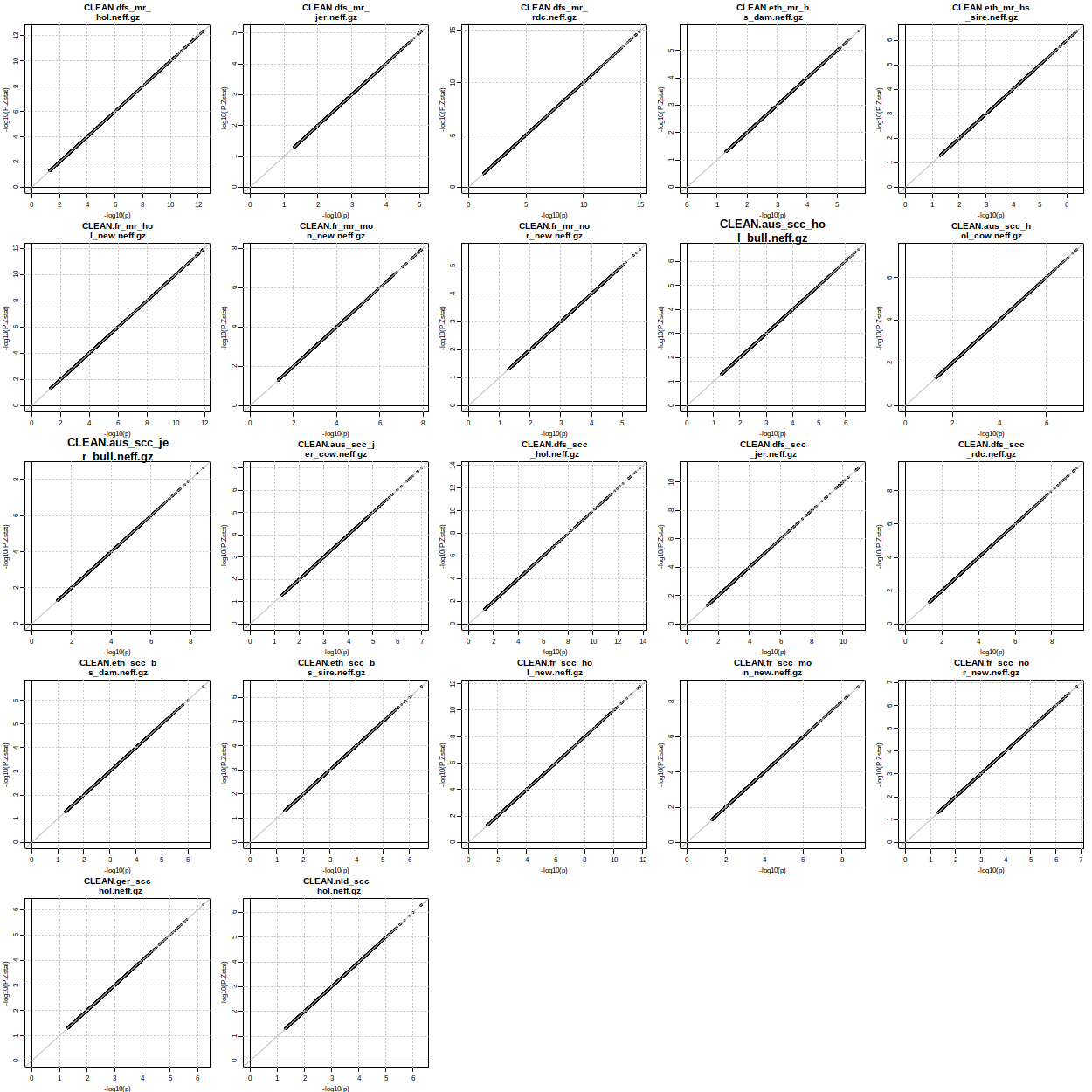


**Figure S3.** **P-Z plot to reveal analytical issues with beta, standard error and P-values.** The filtered dataset showing perfect concordance. The order are: AU clinic mastitis for Holstein bulls, AU clinic mastitis for Jersey bulls, LUKE clinic mastitis for Red bulls, ETH clinic mastitis for Brown Swiss cows, ETH clinic mastitis for Brown Swiss bulls, INRAE clinic mastitis for Holstein bulls, INRAE clinic mastitis for Montbeliarde bulls, INRAE clinic mastitis for Normande bulls, AgVic somatic cell score for Holstein bulls, AgVic somatic cell score for Holstein cows, AgVic somatic cell score for Jersey bulls, AgVic somatic cell score for Jersey cows, AU somatic cell score for Holstein bulls, AU somatic cell score for Jersey bulls, LUKE somatic cell score for Red bulls, ETH somatic cell score for Brown Swiss cows, ETH somatic cell score for Brown Swiss bulls, , INRAE somatic cell score for Holstein bulls, INRAE somatic cell score for Montbeliarde bulls, INRAE somatic cell score for Normande bulls, FBN somatic cell score for Holstein bulls, WUR somatic cell score for Holstein bulls.


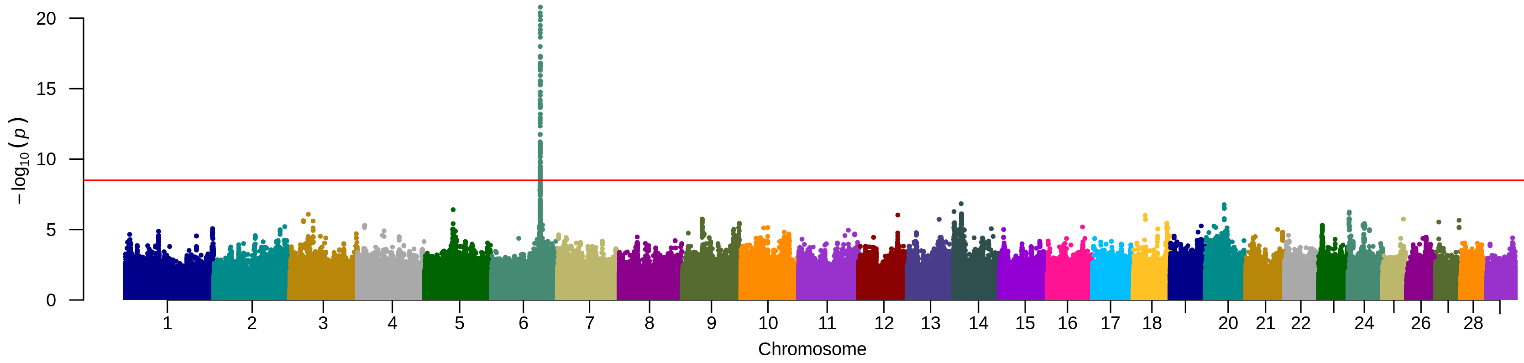


**Figure S4.** **Manhattan plot for meta-analysis of clinic mastitis using METAL software.** The red horizontal line indicates the genome-wide significance level [− log10(p) = 8.5].


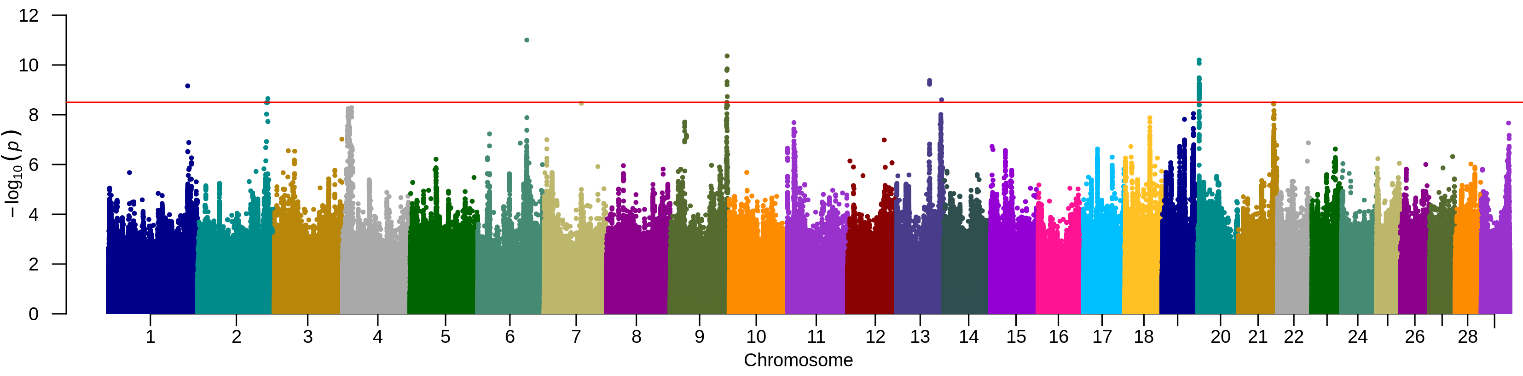


**Figure S5.** **Manhattan plot for meta-analysis of somatic cell score using METAL software.** The red horizontal line indicates the genome-wide significance level [− log10(p) = 8.5].


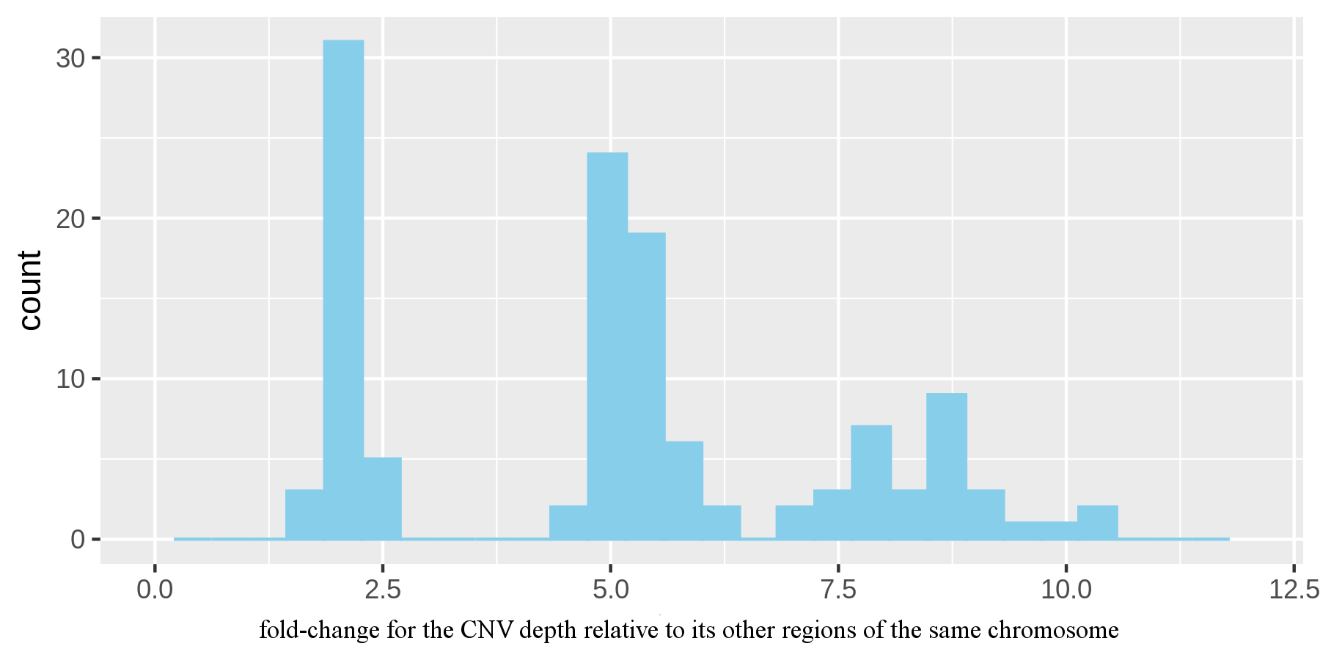


**Figure S6.** **The copy numbers of the GC copy number variant (CNV) in Nordic Holstein animals (123 bulls).** The x-axis is the copy number inferred from the fold-change for the CNV depth relative to other regions of the same chromosome. The y-axis is the number of animals with that copy number.


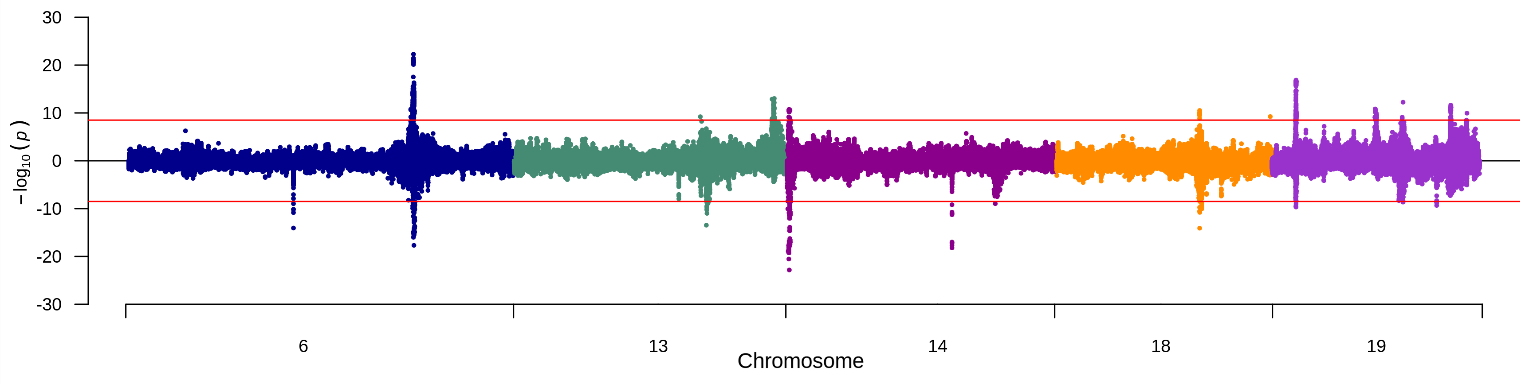


**Figure S7.** **Manhattan plot for meta-analysis of somatic cell score (MR-MEGA_SCS) in the current study and US dataset for the selected chromosomes.** The red horizontal line indicates the genome-wide significance level [− log_10_(p) = 8.5 and -log_10_(p) = -8.5]. Results from current study are plotted as -log10(p) values in the y-axis, while the US GWAS results as log_10_(p) values.
